# A 32-bp deletion in *LiBBX16* underlies the dominant purple leaf trait in crape myrtle

**DOI:** 10.1093/hr/uhag179

**Published:** 2026-04-29

**Authors:** Ping Shen, Lu Feng, Xiufeng Chi, Xin Wang, Zhiting Wan, Qifang Lin, Yiqian Ju, Huan Wang, Yu Han, Tangchun Zheng, Ming Cai, Jia Wang, Tangren Cheng, Qixiang Zhang, Huitang Pan

**Affiliations:** State Key Laboratory of Efficient Production of Forest Resources, Beijing Key Laboratory of Ornamental Plants Germplasm Innovation & Molecular Breeding, National Engineering Research Center for Floriculture, School of Landscape Architecture, Beijing Forestry University, Beijing 100083, China; State Key Laboratory of Efficient Production of Forest Resources, Beijing Key Laboratory of Ornamental Plants Germplasm Innovation & Molecular Breeding, National Engineering Research Center for Floriculture, School of Landscape Architecture, Beijing Forestry University, Beijing 100083, China; State Key Laboratory of Efficient Production of Forest Resources, Beijing Key Laboratory of Ornamental Plants Germplasm Innovation & Molecular Breeding, National Engineering Research Center for Floriculture, School of Landscape Architecture, Beijing Forestry University, Beijing 100083, China; State Key Laboratory of Efficient Production of Forest Resources, Beijing Key Laboratory of Ornamental Plants Germplasm Innovation & Molecular Breeding, National Engineering Research Center for Floriculture, School of Landscape Architecture, Beijing Forestry University, Beijing 100083, China; State Key Laboratory of Efficient Production of Forest Resources, Beijing Key Laboratory of Ornamental Plants Germplasm Innovation & Molecular Breeding, National Engineering Research Center for Floriculture, School of Landscape Architecture, Beijing Forestry University, Beijing 100083, China; State Key Laboratory of Efficient Production of Forest Resources, Beijing Key Laboratory of Ornamental Plants Germplasm Innovation & Molecular Breeding, National Engineering Research Center for Floriculture, School of Landscape Architecture, Beijing Forestry University, Beijing 100083, China; College of Landscape Architecture and Forestry, Shandong Key Laboratory for Germplasm Innovation of Saline-alkaline Tolerant Grasses and Trees, Qingdao Agricultural University, Qingdao 266109, Shandong, China; Institute of Radiation Technology, Beijing Academy of Science and Technology, Beijing 1000875, China; State Key Laboratory of Efficient Production of Forest Resources, Beijing Key Laboratory of Ornamental Plants Germplasm Innovation & Molecular Breeding, National Engineering Research Center for Floriculture, School of Landscape Architecture, Beijing Forestry University, Beijing 100083, China; State Key Laboratory of Efficient Production of Forest Resources, Beijing Key Laboratory of Ornamental Plants Germplasm Innovation & Molecular Breeding, National Engineering Research Center for Floriculture, School of Landscape Architecture, Beijing Forestry University, Beijing 100083, China; State Key Laboratory of Efficient Production of Forest Resources, Beijing Key Laboratory of Ornamental Plants Germplasm Innovation & Molecular Breeding, National Engineering Research Center for Floriculture, School of Landscape Architecture, Beijing Forestry University, Beijing 100083, China; State Key Laboratory of Efficient Production of Forest Resources, Beijing Key Laboratory of Ornamental Plants Germplasm Innovation & Molecular Breeding, National Engineering Research Center for Floriculture, School of Landscape Architecture, Beijing Forestry University, Beijing 100083, China; State Key Laboratory of Efficient Production of Forest Resources, Beijing Key Laboratory of Ornamental Plants Germplasm Innovation & Molecular Breeding, National Engineering Research Center for Floriculture, School of Landscape Architecture, Beijing Forestry University, Beijing 100083, China; State Key Laboratory of Efficient Production of Forest Resources, Beijing Key Laboratory of Ornamental Plants Germplasm Innovation & Molecular Breeding, National Engineering Research Center for Floriculture, School of Landscape Architecture, Beijing Forestry University, Beijing 100083, China; State Key Laboratory of Efficient Production of Forest Resources, Beijing Key Laboratory of Ornamental Plants Germplasm Innovation & Molecular Breeding, National Engineering Research Center for Floriculture, School of Landscape Architecture, Beijing Forestry University, Beijing 100083, China

## Abstract

Structural variations that generate novel alleles are fundamental drivers of phenotypic diversity. In crape myrtle (*Lagerstroemia indica*), an important summer-flowering woody species, the purple leaf trait is prized for its visual appeal and stress resistance, representing a key breeding objective. Although anthocyanin accumulation underlies purple leaf formation, its key genetic locus has remained unclear. Here, we constructed five segregated populations of crape myrtle and confirmed that the purple leaf trait is a single-gene dominant trait. Combining bulked segregant analysis with metabolomics and RNA-seq led to the identification of *LiBBX16*, a B-box zinc-finger transcription factor. This gene has two alleles in purple-leafed (*LiBBX16p* and *LiBBX16a*) and green-leafed (*LiBBX16g* and *LiBBX16a*) plants. *LiBBX16p* contains a unique 32-bp deletion causing a frameshift, premature termination, and nucleo-cytoplasmic localization. An InDel marker derived from this deletion effectively distinguished purple-leafed from green-leafed plants and identified that all plants homozygous for *LiBBX16p* are seedling lethal. Heterologous expression of *LiBBX16p* in *Arabidopsis* resulted in purple leaves and stems, with anthocyanin content ~9.5-fold higher than in controls. Conversely, silencing *LiBBX16* in purple-leafed *L. indica* reduced anthocyanin content, turning leaves green. We further found that the 32-bp deletion enhances LiBBX16’s activation of anthocyanin synthesis-related genes, particularly *LiF3GT* and *LiMYB75*. Mechanistically, unlike LiBBX16g, which directly binds DNA, LiBBX16p likely acts as a coactivator recruited via protein–protein interactions. Overall, our results identified a neomorphic *LiBBX16* allele that coregulates anthocyanin accumulation and seedling development, providing valuable insights into purple leaf formation and a target gene for molecular breeding.

## Introduction

Plant pigments accumulation influences growth, development and reproduction under different environmental conditions [1], enriching phenotypic diversity through leaf color differentiation and improving adaptability to different habitats. Although chlorophyll typically causes leaves to appear green, over-accumulation of anthocyanins results in red or purple leaves. Such brightly colored leaves add a striking touch of color to the urban landscapes during nonflowering seasons, making it more visually appealing. Moreover, as natural antioxidants, anthocyanins can efficiently scavenge free radicals and reactive oxygen species, thereby enhancing plant tolerance to ultraviolet radiation, temperature extremes, drought, and pathogen stress [2–5]. Therefore, elucidating the genetic and molecular mechanisms underlying red and purple leaf formation has become an important direction in plant research.

The formation of most purple leaves results from the extensive accumulation of anthocyanins in the vacuoles of mesophyll cells [6]. The biosynthesis of anthocyanins is accomplished through the phenylpropanoid and flavonoid pathways [7], involving the participation of numerous structural genes [8–10]. The expression of these genes is regulated at multiple levels, with R2R3-MYB transcription factors serving as central regulators that interact with basic helix–loop–helix (bHLH) and WD-repeat (WDR) proteins to form the MBW complex [11–13]. Numerous studies have reported that transcription factors (TFs) associated with the MBW complex that participate in anthocyanin accumulation, including WRKY [14], NAC [15], bZIP [16], ERF [17, 18], several noncoding RNAs [19–21], and BBX family members [22–24].

Natural genetic variation is a key driver of differential anthocyanin accumulation. This variation falls into two main types. The first involves loss-of-function mutations in structural genes of the anthocyanin biosynthesis pathway, including *chalcone synthase* (*CHS*) [25], *flavanone 3-hydroxylase* (*F3H*) [26], *dihydroflavonol 4-reductase* (*DFR*) [27, 28], *anthocyanidin synthase* (*ANS*) [29], and UDP-glucose: *flavonoid 3-O-glucosyltranferase* (*UFGT*) [30], which disrupt pigment synthesis. The second type comprises *cis*-regulatory mutations in MYB TFs that significantly alter pigmentation. For instance, transposon insertions enhance the expression of *MdMYB10*/*MdMYB1* in apple (*Malus × domestica*) [31–34] and *FaMYB10* in strawberry (*Fragaria spp.*) [35], leading to anthocyanin accumulation. Similarly, in tea plant (*Camellia sinensis*), promoter InDels in *CsMYB75* contribute to natural color variation [36, 37]. In pepper (*Capsicum annuum*), coding-sequence variations in *CaMYB113* are responsible for this trait [38]. In tomato (*Solanum lycopersicum*), alternative splicing of *SlAN2like* contributes to anthocyanin diversity [39], while in *Prunus mume*, a large-fragmented inversion cosegregating with the purple leaf trait positions *PmMYB10.5b* at its breakpoint, enhancing anthocyanin accumulation [40]. Beyond MYBs, polymorphisms in other TFs, such as bHLH [41–43], WD40 [44], NAC [45], and BBX [46, 47], also modulate the anthocyanin pathway and contribute to phenotypic diversity.

Crape myrtle (*Lagerstroemia indica*), a prominent summer-flowering woody plant, is highly valued in horticulture due to its ease of propagation, long flowering period, and low maintenance requirements [48, 49]. Purple-leafed cultivars are particularly sought after for their persistent colorful foliage and stress resistance potential, which significantly enhance their ornamental and economic value. However, the genetic mechanism controlling purple leaf formation in *L. indica* remains unclear, posing a challenge for targeted breeding.

Our previous research has confirmed that the purple leaf color in crape myrtle is determined by anthocyanin accumulation (notably cyanidin and pelargonidin derivatives) and identified *LiHY5* and *LiMYB75* as components of the regulatory network [50]. However, the *LiHY5* genotype was not completely consistent with the purple leaf trait in the hybrid population, indicating it was not the major gene controlling purple leaves. This finding prompted us to search for the key genetic locus underlying this economically important trait.

In this study, five hybrid populations with significant leaf color segregation were constructed using ‘Arapahoe’ and ‘Ebony Embers’ as parents, and clarified the inheritance pattern of the purple leaf trait through phenotypic analysis. By integrating bulked segregant analysis (BSA), metabolomics, and transcriptome data, we identified a B-box transcription factor, *LiBBX16*, as the causal gene for the dominant purple leaf trait. We found that a unique 32-bp deletion in *LiBBX16* is responsible for its function: this allele not only acts as a positive regulator of anthocyanin biosynthesis but also leads to seedling lethality in homozygous condition. We further elucidated the molecular mechanism by which this structural variation enhances transactivation of downstream target genes and developed a functional marker for breeding. Our findings thus provide a target gene for molecular breeding of the purple-leafed cultivars in crape myrtle and offer insights into the transcriptional regulation of anthocyanin biosynthesis in purple-leafed plants.

## Results

### Segregation of leaf color trait in hybrid populations of crape myrtle

We used ‘Arapahoe’ and ‘Ebony Embers’ as parents to construct five populations with leaf color segregation (Fig. S1), and the phenotypes of the progenies were shown in Table 1. In Population I, the segregation ratio of purple to green leaves was 1:1 (χ^2^ = 0.013, *P* < 0.05). Subsequently, we selected individuals exhibiting extreme purple and green leaf colors in Population I for hybridization to obtain four additional leaf color segregation populations (Population II, III, IV, V) (Table 1). In Population II, the segregation ratios of purple to green leaves were consistent with both 3:1 (χ^2^ = 0.428, *P* > 0.05) and 2:1 (χ^2^ = 0.643, *P* > 0.05). In Population III, a 1:1 ratio was observed (χ^2^ = 0.034, *P* < 0.05). All hybrid progenies derived from two green-leafed individuals (Population IV) exhibited green leaves exclusively. And in hybrid population of two purple-leafed parents (Population V), a segregation ratio of 2:1 was observed, aligning with a genetic model indicative of dominant homozygous lethality (χ^2^ = 0.36，*P* < 0.05). The results suggested that purple leaf trait in *L. indica* is dominant relative to green leaf trait, and the purple leaf trait is controlled by a single gene, and there may be a dominant homozygous lethal phenomenon.

**Table 1:** The leaf color of the progenies of different populations.

Population	Female parent	Male parent	Leaf color of female parent	Leaf color of male parent	Number of purple-leaf progenies	Number of green-leaf progenies	Expected segregation ratio
I	‘Arapahoe’	‘Ebony Embers’	Green leaf	Purple leaf	40	39	1:1
II	Progeny No. 23 in Population I	Progeny No. 29 in Population I	Purple leaf	Purple leaf	45	18	3:1/2:1
III	Progeny No. 80 in Population I	Progeny No. 29 in Population I	Green leaf	Purple leaf	60	58	1:1
IV	Progeny No. 80 in Population I	Progeny No. 37 in Population I	Green leaf	Green leaf	0	16	
V	‘Ebony Embers’	Progeny No. 32 in Population I	Purple leaf	Purple leaf	109	60	2:1

### BSA analysis of purple leaf trait in crape myrtle

To identify the key genetic locus controlling the purple leaf trait in *L. indica*, two parent pools (‘Arapahoe’ (M54) and ‘Ebony Embers’ (BD4)) and two progeny pools with extreme leaf color (PL-F1 for purple leaf pool and GL-F1 for green leaf pool) in Population I were constructed for BSA-seq. The pools of BD4, M54, PL-F1 and GL-F1 generated 12 612 521 400-, 13 591 428 000-, 10 889 805 300-, and 11 377 505 400-bp high-quality raw reads, respectively (GC rate ≥40.87%, Q20 ≥94.76%, Q30 ≥87.02%) (Table S1). After filtering each sample pool, the sequencing depth of the four pools was >34×, the alignment rate with *L. indica* reference genome was between 91.79% and 93.48%, and the coverage rate of 10× was >82% (Table S2). Finally, a total of 2.864 million single-nucleotide polymorphisms (SNPs) and 322 642 insertions or deletions (InDels) were detected in BD4 pool, 3.889 million SNPs and 443 113 InDels were detected in M54 pool, 4.249 million SNPs and 474 019 InDels were detected in PL-F1 pool, and 4.323 million SNPs and 482 837 InDels were detected in GL-F1 pool (Tables S3 and S4). After eliminating the low-quality variants, 2 041 428 variants were identified across all chromosomes (Dataset S1).

**Figure 1:**
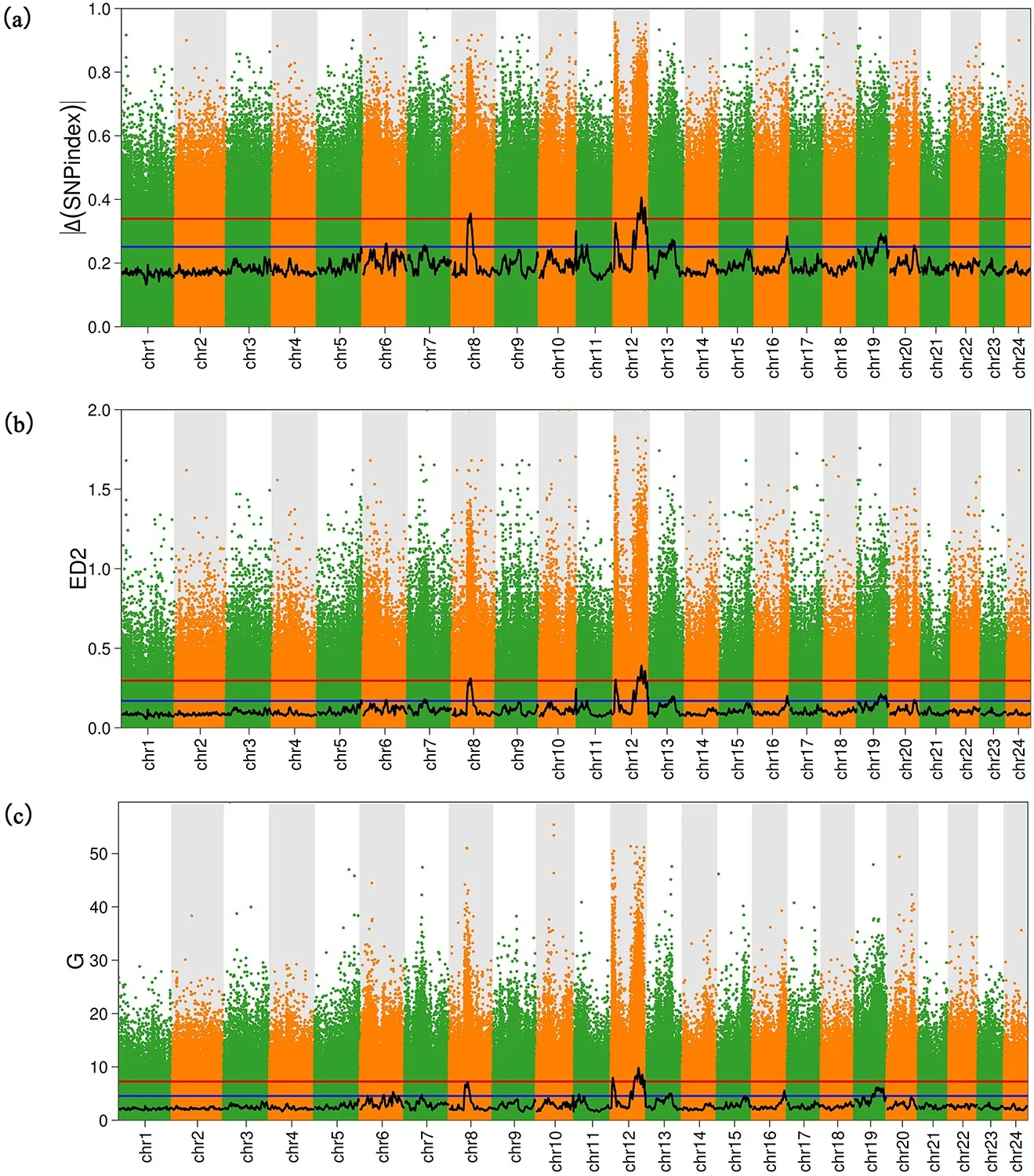
The distribution of the values obtained by three BSA analysis methods on the chromosomes. (a) Scatter diagram of the ΔSNP index. The black line indicates the fitted ΔSNP index value, and the blue and red lines represent thresholds of 95% and 99%, respectively. (b) Scatter diagram of ED^2. The black line indicates the fitted ED^2 value. (c) Scatter diagram of the G-statistic. The black line indicates the fitted G value. The blue and red lines in the figure represent 95% and 99% thresholds, respectively.

The SNP index and the delta SNP index were calculated according to the SNP and InDel mutation sites determined by the four pools and the sliding window method (taking the average value in the window as the fitted value, 500 kb as the window size, and 10 kb as the step size). The SNP index and the delta SNP index were mapped with the genome position, and the candidate intervals were located between 5 930 001 and 7 270 000 bp on chromosome 8 and 8 450 001 and 11 890 000 bp on chromosome 12, with physical distances of 1.34 and 3.44 Mb, respectively (99% confidence threshold, Fig. 1a and Table S5). Three regions were obtained by ED algorithm, which were located in the 0.92-Mb region of 6 290 001–7 210 000 bp on chromosome 8, 0.63-Mb region of 440 001–1 070 000 bp on chromosome 12, and 3.44-Mb region of 8 480 001–11 920 000 bp, respectively (99% confidence threshold, Fig. 1b and Table S6). In addition, three loci were also mapped on chromosome 12 by using the G-statistic method, which were in the 0.83-Mb region of 400 001–1230 000 bp, in the 3.65-Mb region of 8 270 001–11 920 000 bp, and in the 0.56-Mb region of 11 970 001–12 530 000 bp, respectively (99% confidence threshold, Fig. 1c and Table S7). The regions identified by the three methods showed overlap. Considering the high heterozygosity of woody plants, we defined the union of the three results as the candidate interval, which was located on chromosome 8 and chromosome 12 (Table S8), and 481 genes were annotated for subsequent analysis (Dataset S2).

### Identify the important genes involved in purple leaf formation in crape myrtle

Using the previous transcriptome data obtained in the early stage [50]，the genes with no functional annotation and FPKM <10 during the purple and green leaf development stages were excluded from 481 genes, and 220 candidate genes were retained (Dataset S2). According to the targeted metabolomics and transcriptomics data obtained in the previous study [50], Pearson correlation analysis was performed between the differentially expressed genes screened in RNA-seq and the anthocyanin content in leaves, and 1088 isoforms (correlation coefficient *R*^2^ ≥ 0.9 and *P* ≤ 0.05) were obtained (Dataset S4). After reference-based alignment, a total of 931 candidate genes were identified.

The intersection of 220 genes and 931 genes was performed, and 10 candidate genes were retained (Table 2). Among them, there were two genes that may be involved in the flavonoid biosynthesis pathway, namely *Lin_chr8_0539* and *Lin_chr12_0051*. *Lin_chr8_0539* is a structural gene in the anthocyanin biosynthesis pathway, annotated as an anthocyanin synthase, and named *LiLDOX*. *Lin_chr12_0051* is a zinc finger transcription factor with a B-box domain and belongs to the BBX family protein. It may be involved in light-induced anthocyanin biosynthesis and is named *LiBBX16*.

**Table 2:** Candidate genes in purple leaf formation identified by association analysis of targeted metabolomics, RNA-Seq, and BSA-Seq data.

**Gene ID**	**Peak**	**Symbol**	**Description**	**TF_family**
Lin_chr8_0445	0.36	MO3	XP_031400198.1 monooxygenase 3 isoform X1 [*Punica granatum*]	
Lin_chr8_0474	0.36	SEC16B	XP_031376778.1 protein transport protein SEC16A homolog [*P. granatum*]	
Lin_chr8_0494	0.36	TDX	CAN68309.1 hypothetical protein VITISV_043507 [*Vitis vinifera*]	
Lin_chr8_0498	0.36	CYP75A5	PHT58079.1 hypothetical protein CQW23_00442 [*Capsicum baccatum*]	
Lin_chr8_0539	0.36	LDOX	QJS57392.1 anthocyanidin synthase [*Lagerstroemia indica*]	
Lin_chr12_0051	7.98	BBX	OWM66929.1 hypothetical protein CDL15_Pgr008098 [*P. granatum*]	DBB
Lin_chr12_1158	0.41		XP_031404273.1 uncharacterized protein LOC116213459 [*P. granatum*]	-
Lin_chr12_1072	0.41	ERD4	OWM63837.1 hypothetical protein CDL15_Pgr006099 [*P. granatum*]	
Lin_chr12_1076	0.41	PSAK	XP_031405365.1 photosystem I reaction center subunit psaK, chloroplastic [*P. granatum*]	

### Variations in the coding and promoter regions of *LiLDOX* are not related to purple leaf

Structural gene *LiLDOX* plays a crucial role in anthocyanin biosynthesis. To investigate whether the purple leaf trait in crape myrtle is caused by coding sequence (CDS) variations in *LiLDOX*, we cloned its full-length CDS. The results revealed that *LiLDOXp* and *LiLDOXa* were present in purple-leafed progeny, whereas *LiLDOXg* and *LiLDOXa* were found in green-leafed progeny. Amino acid sequence alignment revealed that all three proteins consist of 355 amino acids and contain the conserved 2-oxoglutarate Fe (II) oxygenase domain. LiLDOXp and LiLDOXg differed at only three amino acid positions (Fig. S2). Subsequently, we randomly selected nine purple-leafed and nine green-leafed individuals from Population I and cloned the CDSs of *LiLDOX* separately. Genotyping of these individuals showed that the three missense mutations in *LiLDOX* did not cosegregate with leaf-color traits in the hybrid population (Table 3). Therefore, sequence variations in the coding region of *LiLDOX* are not the direct cause of purple leaves in crape myrtle.

**Table 3:** Amino acid substitutions resulting from missense mutations in LiLDOX among purple- and green-leafed progeny from the ‘Arapahoe’ × ‘Ebony Embers’ population.

**Progeny ID**	**Leaf color**	**Mutational sites**		
		**R55K**	**D102E**	**S187T**
25	Green	−	−	−
35	Green	−	−	−
36	Green	−	−	−
40	Green	−	−	−
43	Green	−	+	−
46	Green	−	+	−
50	Green	−	+	−
59	Green	−	+	−
63	Green	−	−	−
1	Purple	+	+	+
2	Purple	+	+	+
5	Purple	+	+	+
6	Purple	−	−	−
9	Purple	−	−	−
16	Purple	−	−	−
17	Purple	−	−	−
29	Purple	−	−	−
30	Purple	−	−	−

Note: R55K indicates a substitution from arginine (R) to lysine (K) at position 55; D102E, aspartic acid (D) to glutamic acid (E) at position 102; and S187T, serine (S) to threonine (T) at position 187. ‘+’ indicates a missense mutation；‘–’ indicates the wild-type sequence.

To determine whether the purple leaf trait is related to sequence variation in the *LiLDOX* promoter, we cloned a 1806-bp fragment upstream of its ATG start codon. The promoter sequences were identical between purple- and green-leafed progeny(Fig. S3a). Bioinformatic analysis using PlantCARE identified multiple *cis*-regulatory elements (Fig. S3b), including 11 MYB/bHLH-binding sites (containing MBSI), 12 light-responsive elements, 14 hormone-responsive elements (associated with jasmonic acid, abscisic acid, and gibberellin), 10 stress-responsive elements (related to mechanical injury, low temperature, drought, and hypoxia), and 1 element associated with meristem expression. These findings suggested that *LiLDOX* expression may be regulated by light signaling and multiple hormones, and may participate in hypoxia adaptation, meristem development, and response to various abiotic stresses.

Based on these findings, variations in its coding and promoter regions were excluded as the direct causes of purple leaf formation in crape myrtle. Previous evidence indicated that the purple leaf trait in crape myrtle is governed by a single gene. Given that anthocyanin accumulation is regulated at the transcriptional level rather than by the expression level of any single structural gene [50], we selected *LiBBX16* as the key candidate gene for regulating this trait in crape myrtle.

### Cosegregation of 32-bp deletion in the *LiBBX16* gene and purple leaf trait of crape myrtle

**Figure 2:**
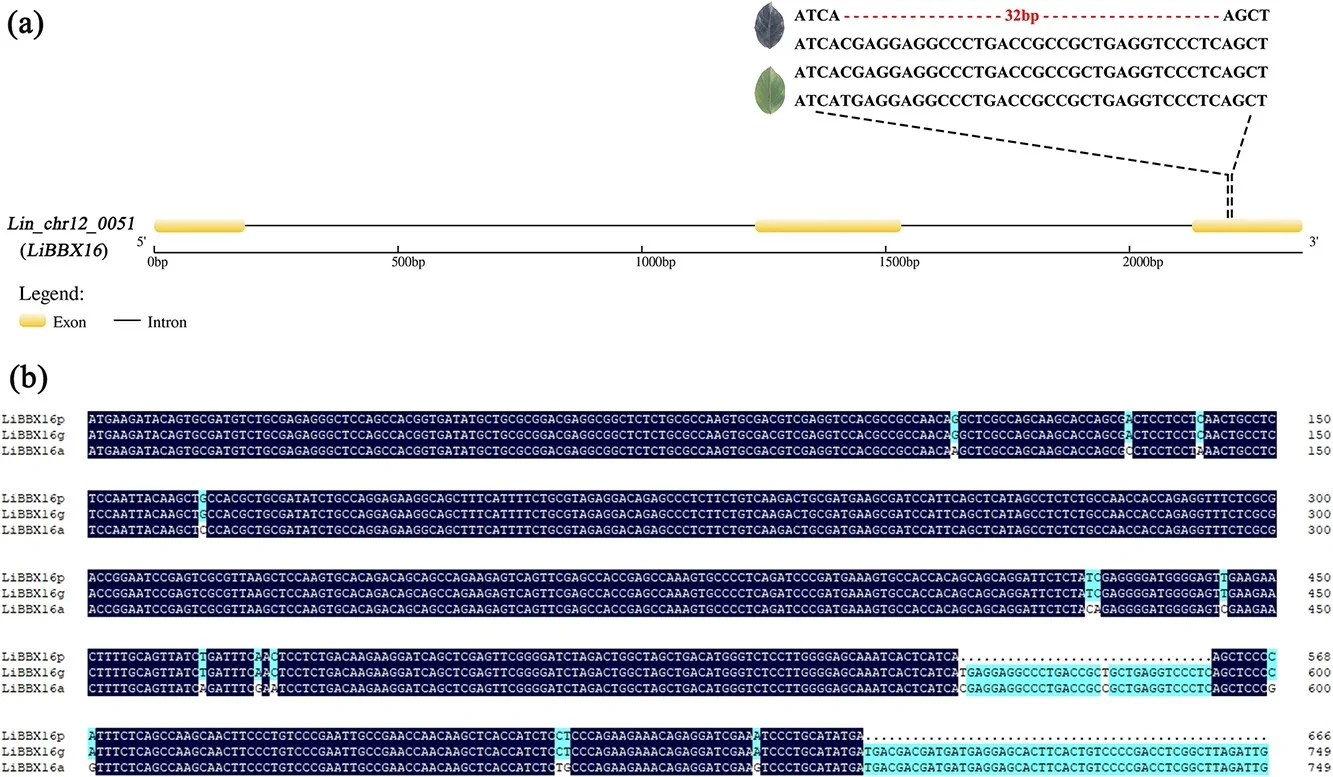
Gene structure and sequence of *LiBBX16*. (a) Gene structure and deletion of *LiBBX16*. (b) Coding sequence alignment of *LiBBX16p, LiBBX16g*, and *LiBBX16a. LiBBX16a* is present in both purple- and green-leafed progenies, whereas *LiBBX16g* and *LiBBX16p* are specific to green- and purple-leafed progenies, respectively.

To confirm the potential involvement of *LiBBX16* in purple leaf formation, we cloned the genomic DNA and CDS of *LiBBX16* from both purple- and green-leafed progenies. The results showed that *LiBBX16* has two alleles: *LiBBX16p* and *LiBBX16a* were identified in purple-leafed progenies, while *LiBBX16g* and *LiBBX16a* were found in green-leafed progenies. Notably, compared with *LiBBX16g* and *LiBBX16a*, *LiBBX16p* had a 32-bp deletion located in the third exon (Fig. 2a and b), indicating that this variation may be exclusive to purple-leafed individuals of *L. indica*.

To characterize the expression regulation of *LiBBX16* further, we cloned its promoter region. The promoter sequences of *LiBBX16* from both purple- and green-leafed progeny were identical (Fig. S4a). Using PlantCARE software, we identified multiple *cis*-regulatory elements, including 16 light-responsive elements, 3 growth-and-development regulatory elements, 19 hormone-responsive elements (associated with abscisic acid, jasmonic acid, and auxin), and 8 stress-responsive elements (related to anaerobic induction, drought, high salt, and low temperature) (Fig. S4b). These findings suggested that *LiBBX16* is responsive to light, multiple hormones and stress factors while also involved in regulating plant meristem expression.

**Figure 3:**
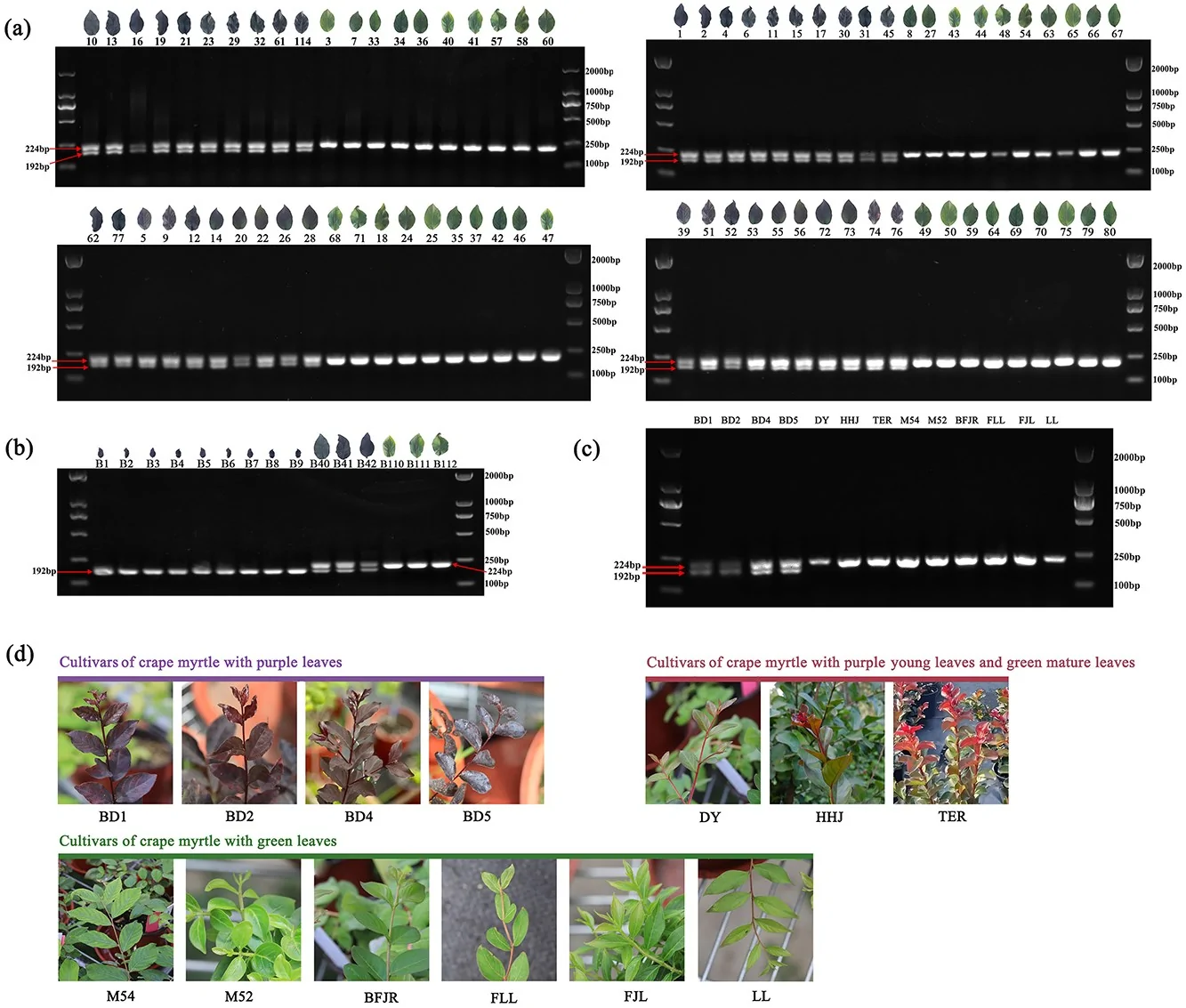
PCR amplification results of the *LiBBX16* gene fragment obtained using primers developed based on the 32-bp deletion in purple- and green-leafed crape myrtle. (a) The PCR amplification results of the *LiBBX16* gene fragment obtained in progenies with different leaf colors in Population I. (b) The PCR amplification results of the *LiBBX16* gene fragment obtained in progenies of Population V, including nine progenies with purple leaves and extremely short plant type (B1–B9), three purple-leaf progenies (B40–B42), and three green-leaved progenies (B110–112). (c) The PCR amplification results of the *LiBBX16* gene fragment obtained in 13 cultivars of *L. indica*, which is listed in Table S9. (d) Leaf colors of 13 crape myrtle cultivars.

To verify whether the leaf colors are cosegregated with the 32-bp deletion in *LiBBX16* in the hybrid populations, primers were designed targeting this deletion region for PCR amplification (Fig. S5). In Population I, electrophoresis of 40 purple-leafed progenies contained two bands (224 and 192 bp), while 39 green-leafed progenies contained only one band (224 bp) (Fig. 3a). Interestingly, we found a type of individuals characterized by extremely short stature and purple-black leaves, which was called ‘mini violet’ in this study. These individuals exhibit very few and short roots, and they usually die sequentially within a year of growth (Fig. S6). The electrophoresis results of nine ‘mini violet’ plants contained only a single band at 192 bp (Fig. 3b). The results revealed that the deletion in *LiBBX16* exists only in purple-leafed progenies. To further investigate whether there is a similar deletion in different cultivars of *L. indica*, we analyzed 13 cultivars with three leaf color phenotypes (Fig. 3d). The results showed that two bands (192 and 224 bp) were found in the purple-leafed cultivars, while only one band (224 bp) was observed in cultivars with purple young leaves and green mature leaves, as well as in green-leafed cultivars (Fig. 3c). The results further proved that the 32-bp deletion in *LiBBX16* is cosegregated with purple leaf trait in *L. indica*.

### Structural and functional analysis of LiBBX16 and its deletion mutation

**Figure 4:**
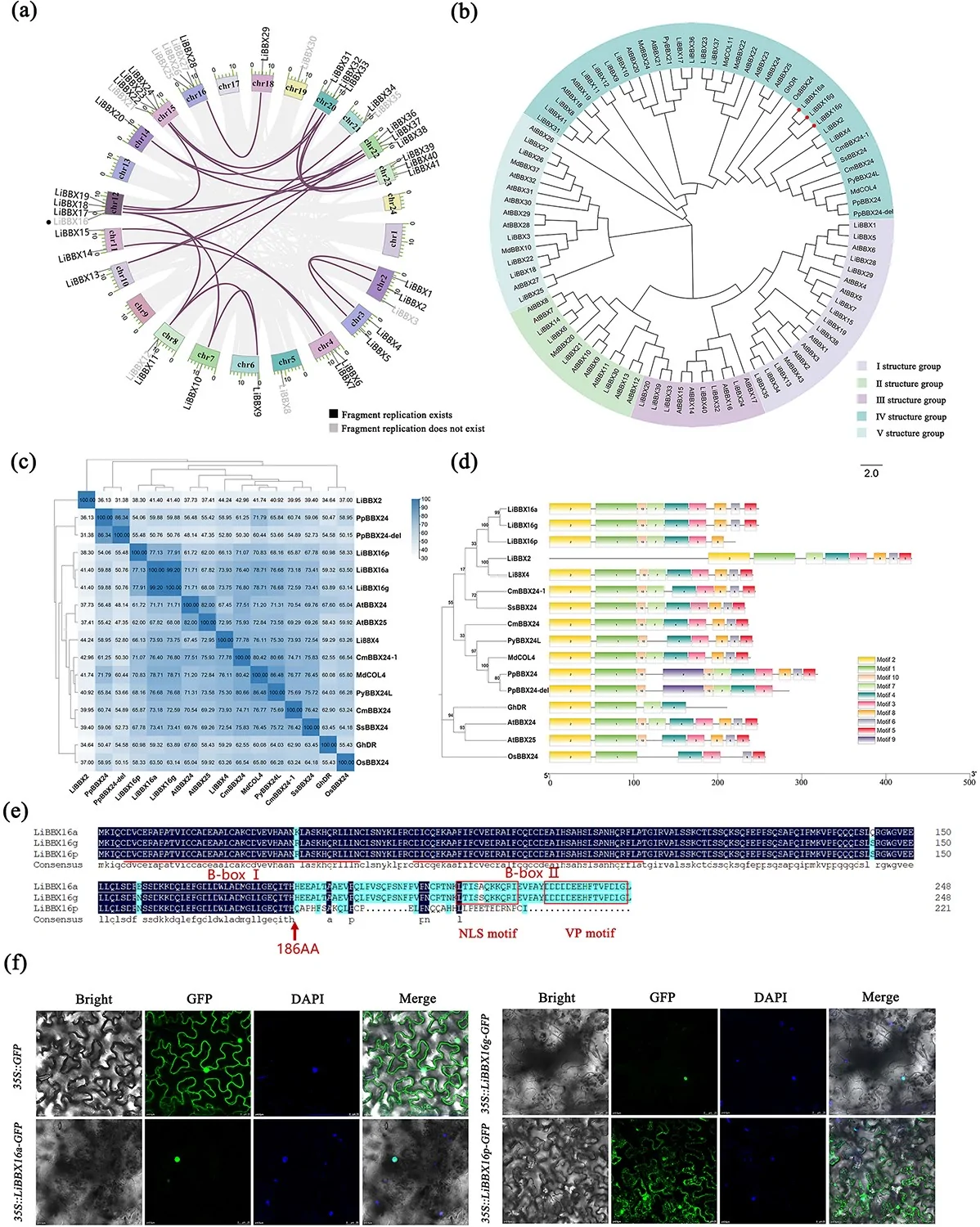
Intraspecific collinearity analysis, phylogenetic relationships, sequence characteristics, and subcellular localization of three genotypes of LiBBX16. (a) Intraspecific collinearity analysis of BBX family in *L. indica.* (b) Phylogenetic tree of BBX proteins in *L. indica*, *A. thaliana*, and other species. Plant species and GenBank accession numbers are listed in Table S11. (c) The amino acid sequence similarity matrix of BBX24 and BBX25 branches. (d) Conserved motif analysis of BBX24 and BBX25 branches. (e) Alignment of the amino acid sequences of LiBBX16a, LiBBX16g, and LiBBX16p. LiBBX16p lost the NLS and VP motifs enclosed in red rectangles due to the 32-bp deletion. (f) Subcellular localization of LiBBX16a, LiBBX16g, and LiBBX16p in *N. benthamiana* cells. Nuclear localization was detected by staining with 4′,6-diamidino-2-phenylindole (DAPI).

To explore whether the candidate gene exhibits functional differentiation or redundancy with other BBX members, we identified 41 BBX genes from the genome of *L. indica*. Based on their chromosomal distribution, these genes were designated as *LiBBX1*-*LiBBX41*, with *LiBBX16* located on chromosome 12 (Fig. S7). Intraspecific collinearity analysis revealed that *LiBBX16* exists as a single copy in *L. indica*, without fragment replication occurring (Fig. 4a). Phylogenetic analysis indicated that LiBBX16 were clustered with homologs of the *Arabidopsis* BBX family structure in group IV, specifically AtBBX24 and AtBBX25. Additionally, GhDR and PpBBX24-del, which have been reported to be related to anthocyanin accumulation, were also found in this branch (Fig. 4b). Sequence similarity analysis showed that the similarity index among genes within this branch ranged from 31% to 99%, and the similarity index between LiBBX16a, LiBBX16g, LiBBX16p and AtBBX24, GhDR, and PpBBX24-del varied between 51% and 99% (Fig. 4c). Conserved motif analysis revealed that protein motifs within this branch were similar. Notably, LiBBX16p, GhDR, and PpBBX24-del lacked Motif 5 and Motif 6 (Fig. 4d).

Structural analysis of LiBBX16 revealed that it contained two B-box domains (B-box I and B-box II) in the N-terminus, and a nuclear localization signal (NLS) motif and a valine-proline (VP) motif in the C-terminus. It is noteworthy that LiBBX16p contains a 32-bp deletion, which advances the termination codon and leads to the absence of two motifs (Fig. 4e). LiBBX16a and LiBBX16g have the same sequence length, with only amino acid changes at four sites (Fig. 4e). Through the analysis using the PredictProtein website, it was found that the secondary structures of the three proteins comprised random coil, extended chain, and α-helix, with random coil representing the largest proportion. ProtParam analysis indicated that all the three proteins were acidic, hydrophilic, and unstable, and LiBBX16p was slightly more stable (Table S10). TMHMM analysis confirmed that none of the three proteins were classified as transmembrane proteins.

Compared with LiBBX16a and LiBBX16g, the N-terminal region of LiBBX16p lacks the NLS motif due to the early translation termination caused by 32-bp deletion. Consequently, it is speculated that the subcellular localization pattern of LiBBX16p may be different. The GFP fusion vectors for the LiBBX16 protein were constructed and transiently expressed in *Nicotiana benthamiana* leaves. It was found that the fluorescent signals of both LiBBX16a and LiBBX16g predominantly localized in the nucleus, while the fluorescence of LiBBX16p was observed in both the nucleus and cytoplasm simultaneously (Fig. 4f). The results suggested that 32-bp deletion affects the localization of LiBBX16 protein within plant cells, which may lead to distinct functional roles of LiBBX16 in different genotypes.

In addition to altering subcellular localization, the 32-bp deletion also modulates the intrinsic transcriptional activation activity of LiBBX16. Transcriptional activation assays revealed that the C-terminal fragment containing the 32-bp deletion (BD-LiBBX16p-C) exhibited strong self-activation activity, whereas the C-terminal (BD-LiBBX16g-C) and N-terminal (BD-LiBBX16-N) fragments of LiBBX16g showed no such activity (Fig. S8), indicating that the 32-bp deletion generates a novel transcriptional activation domain in the C-terminus. Notably, neither full-length protein (BD-LiBBX16p nor BD-LiBBX16g) exhibited self-activation activity.

### *LiBBX16p* induces anthocyanin biosynthesis in leaves of crape myrtle and *Arabidopsis*

**Figure 5:**
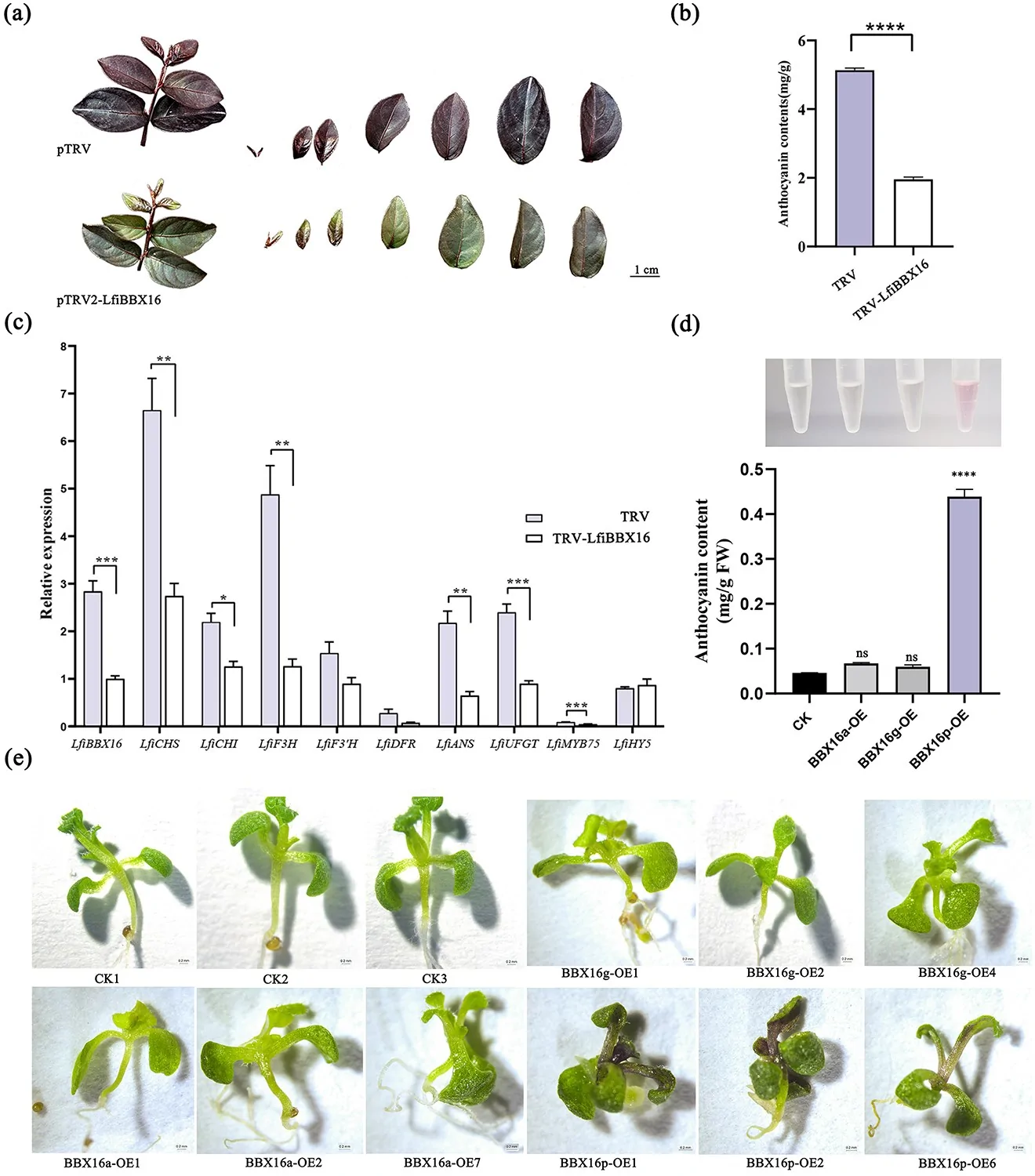
The function of *LiBBX16* in regulating anthocyanin synthesis in leaves. (a) The changes in the color of new leaves of crape myrtle after 4 weeks of treatment with TRV and TRV2-*LiBBX16*. (b) Total anthocyanin contents in crape myrtle leaves after 4 weeks of treatment with TRV and TRV2-*LiBBX16*. (c) Changes of relative expression levels of *LiBBX16* and anthocyanin structural genes in crape myrtle leaves after silencing *LiBBX16*. Error bars indicate the SDs of five biological replicates. Different lowercase letters indicate significance level at *P* < 0.05 by one-way ANOVA. ^*^ indicates significant differences at level of *P* < 0.05, ^**^ indicates significant differences at level of *P* < 0.01, and ^***^ indicates significant differences at level of *P* < 0.001 by Student’s *t*-test. (d) Total anthocyanin contents of *Arabidopsis* seedlings transformed respectively by empty vector, *LiBBX16a*, *LiBBX16g*, and *LiBBX16p*. CK represents transgenic empty vector, used as the control. OE represents overexpression. (e) The phenotypes of 10-day-old *Arabidopsis* seedlings transformed respectively by empty vector, *LiBBX24a*, *LiBBX24g*, and *LiBBX24p*.

To verify the functions of *LiBBX16* in purple leaf formation, we employed a virus-induced gene silencing (VIGS) assay to transiently silence *LiBBX16* in purple-leafed crape myrtle. Since it is challenging to separately silence the three alleles of LiBBX16 using VIGS, a common fragment was selected from all the three alleles to assess its function. Three weeks after VIGS treatment, new leaves emerged from the pruned branches, and no significant phenotypic changes were observed in either the control or wild-type plants. In contrast, after being treated with pTRV2-*LiBBX16*, the purple color of leaves in the new shoot was reduced (Fig. 5a). Meanwhile, the anthocyanin content dropped to ~61.8% of the control (Fig. 5b). Concurrently, the expression level of *LiBBX16* decreased to 64.8%. Silencing of the *LiBBX16* gene led to a decrease in the expression levels of *LiCHS*, *LiCHI*, *LiF3H*, *LiF3′H*, *LiDFR*, *LiANS*, and *LiUFGT* to 41.8%–74.1%, and the expression level of *LiMYB75* also decreased to 46.2%. However, the expression level of *LiHY5* was not affected (Fig. 5c).

To further elucidate functional differences among three alleles of *LiBBX16*, the 35S::LiBBX16a/p/g-GFP construct were separately transformed into *Arabidopsis* Col-0 to obtain T1 transgenic lines. After 10 days of cultivation, plants with two to four true leaves were transplanted. Compared with the transgenic lines overexpressing empty vector (CK), there was no significant difference in leaf color between the transgenic lines overexpressing *LiBBX16a* and *LiBBX16g*. However, the leaves and stems of transgenic lines overexpressing *LiBBX16p* appeared purple (Fig. 5e), with total anthocyanin content ~9.5 times than that of CK (Fig. 5d). The results indicated that *LiBBX16p* positively regulates anthocyanin accumulation in leaves, leading to the formation of purple leaves. Furthermore, since neither *LiBBX16a* nor *LiBBX16g* positively regulates anthocyanin accumulation and both lack the 32-bp deletion, which is the major genetic locus of the purple leaf trait in *L. indica* (Fig. 4f), we regard *LiBBX16a* and *LiBBX16g* as one gene for subsequent experiments.

### Potential target genes of LiBBX16p associated with purple leaf formation

**Figure 6:**
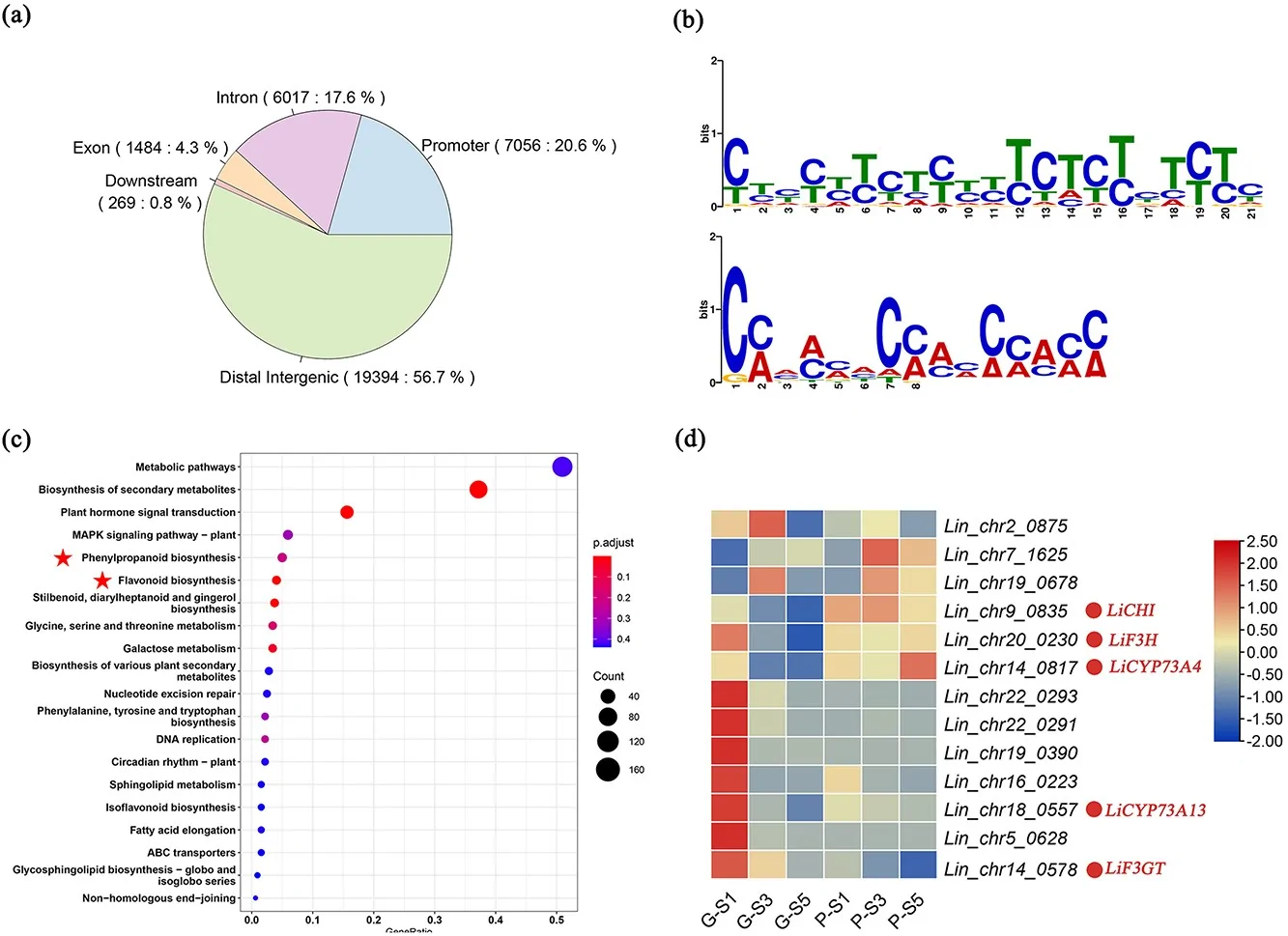
Association analysis of DAP-seq and RNA-seq data to screen the downstream target genes of *LiBBX16p*. (a) Distribution of LiBBX16p-binding regions in *L. indica* genome based on DAP-seq analysis. (b) Conserved motifs of LiBBX16p identified from the DAP-seq data. (c) KEGG enrichment analysis of candidate genes obtained by Dap-seq and RNA-seq association analysis. (d) The heat map of candidate genes in flavonoid biosynthesis pathway was analyzed by KEGG analysis.

To determine the regulatory role of *LiBBX16* in purple leaf formation, DAP-seq was conducted. A total of 34 220 *LiBBX16p* binding sites were identified, of which the majority were located in intergenic (56.7%) and promoter (20.6%) regions, and to a lesser extent in intronic (17.6%) and exonic (4.3%) regions (Fig. 6a). Through MEME-CHIP analysis of binding peaks, two motif sequences with the most significant enrichment were identified: C[C/T][C/T][C/T][C/T][C/T]CT[C/T][C/T][C/T][C/T]CTC[C/T][C/T]T[C/T][C/T]C and C[A/C][A/C][A/C][A/C][A/C/T]C[A/C][A/C][A/C]C[A/C]A[A/C][A/C] (Fig. 6b).

We focused on the peak-related genes annotated as promoters. By integrating the transcriptome data [50], we intersected the differentially expressed genes with the genes related to the promoter identified by DAP-seq, and obtained a set of 1994 genes (Fig. S9). After removing the genes with no functional annotation and FPKM ≤10 during leaf development stages, we obtained 1140 genes (Dataset S5). Kyoto Encyclopedia of Genes and Genomes (KEGG) pathway enrichment analysis on the 1140 genes showed that the top six enriched pathways included metabolic pathways, biosynthesis of secondary metabolites, plant hormone signal transduction, MAPK signaling pathway, phenylpropanoid biosynthesis, and flavonoid biosynthesis pathway (Fig. 6c), indicating *LiBBX16p* may interact with multiple biological pathways. The analysis of these pathways revealed that a total of 14 genes were enriched in flavonoid biosynthesis, flavone and flavonol biosynthesis, and anthocyanin biosynthesis pathways. Based on the functional annotation and expression differences (Fig. 6d and Table S12), we found five genes related to anthocyanin synthesis, including 2 cinnamate-4-hydroxylase (*C4H*), chalcone isomerase (*CHI*), *F3H*, and flavonoid 3-glucosyltransferase (*F3GT*). In the phenylpropanoid biosynthesis pathway, two *C4H* genes were found in the upstream gene group: *Lin_chr14_0817* (named *LiCYP73A4*) and *Lin_chr18_0557* (named *LiCYP73A13*), which catalyze reactions that provide essential precursor substances for anthocyanin synthesis and represent both a foundational and rate-limiting step in this pathway. In the flavonoid biosynthesis pathway, *Lin_chr9_0835* (named *LiCHI*), *Lin_chr20_0230* (named *LiF3H*), and *Lin_chr14_0578* (named *LiF3GT*) were found. DAP-Seq analysis further confirmed that the promoter regions of the five genes all exhibited significant and specific binding peaks for LiBBX16p, which were consistently higher than control levels across biological replicates (Fig. S10), supporting their status as direct transcriptional targets.

BBX protein regulates anthocyanin synthesis primarily through two mechanisms: one is to directly activate or inhibit the expression of anthocyanin structural genes; the second is to coordinate or individually target key TFs such as MYB, thereby indirectly regulating the entire metabolic pathway [22, 23]. In our study, two MYBs with R2R3 domains from Dap-seq data were obtained (Table S13 and Fig. S10), including *Lin_chr13_0470* (named *LiMYB75*) and *Lin_chr19_0277* (named *LiMYB5*). It is speculated that *LiBBX16p* may indirectly participate in the regulation of anthocyanin in leaves of *L. indica* by binding to the promoter regions of *LiMYB75*and *LiMYB5*, thereby inducing their upregulation.

### Allelic variation in *LiBBX16* affects the expression of key genes involved in anthocyanin synthesis to cause anthocyanin accumulation in leaves

**Figure 7:**
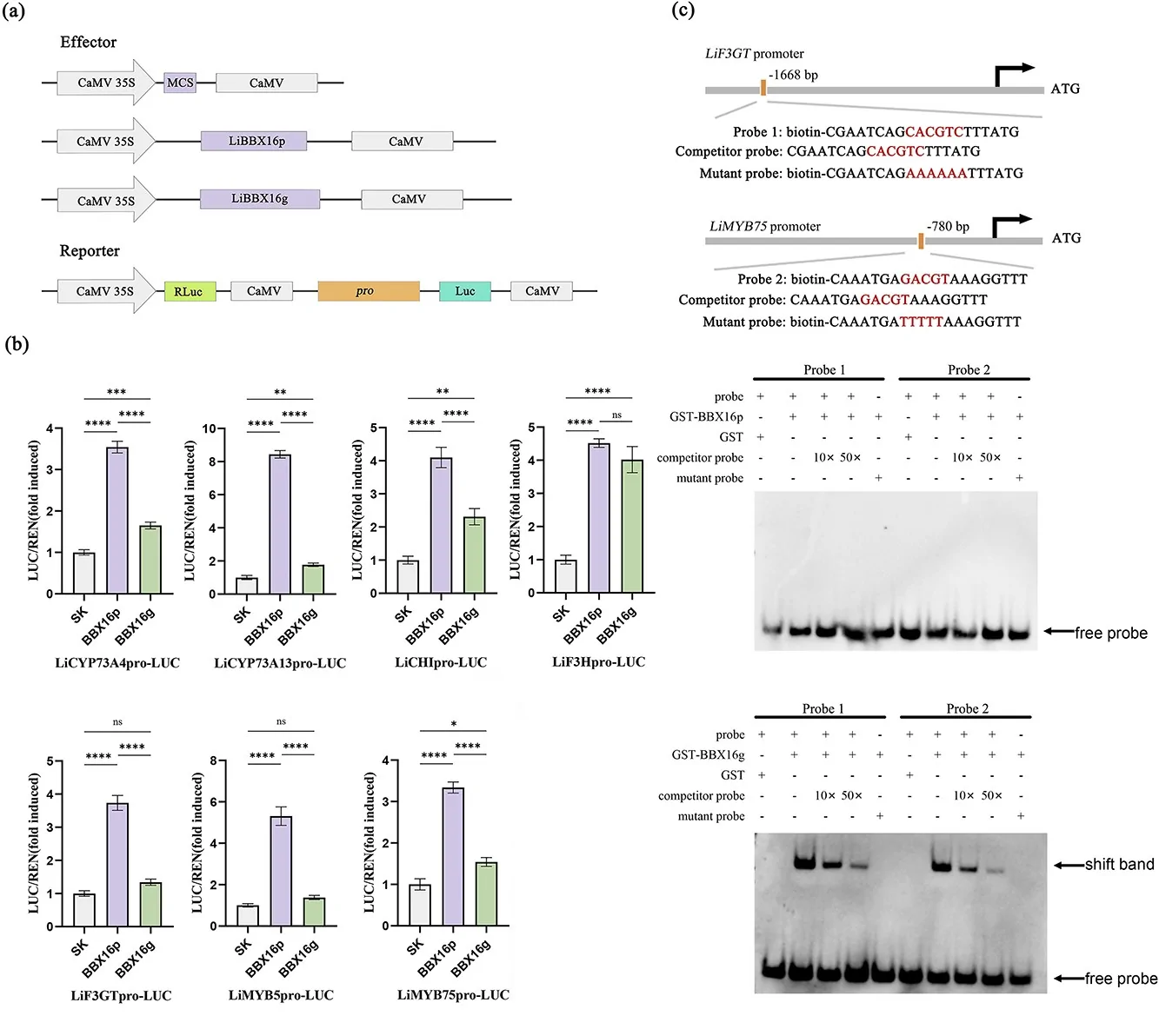
The regulatory effects of LiBBX16p and LiBBX16g on anthocyanin pathway genes*.* (a) LiBBX16p and LiBBX16g were inserted into 35S-pGreenII 62-SK vector, with the empty vector as control, and the promoters of *LiCYP73A4*, *LiCYP73A13*, *LiCHI*, *LiF3GT, LiMYB5*, and *LiMYB75* were inserted into pGreenII 0800-LUC vector, respectively. (b) Effects of *LiBBX16p* and *LiBBX16g* on the promoter activity of *LiCYP73A4*, *LiCYP73A13*, *LiCHI*, *LiF3GT, LiMYB5*, and *LiMYB75* by dual-luciferase assay. Error bars indicate the SDs of nine biological replicates. Different lowercase letters indicate significance level at *P* < 0.05 by one-way ANOVA. ^*^ indicates significant differences at level of *P* < 0.05, ^**^ indicates significant differences at level of *P* < 0.01, and ^***^ indicates significant differences at level of *P* < 0.001 by Student’s *t*-test. (c) EMSA verified the binding of GST-BBX16p and GST-BBX16g fusion proteins to the G-box element in the *LiF3GT* promoter and the G-box-like elements in the *LiMYB75* promoter, respectively. ‘–’ and ‘+’ indicate the absence and presence of the corresponding probe or protein. ‘10×’ and ‘50×’ indicate the ratios of the competitor probe to the corresponding the biotin-labeled G-box(−like) probe, respectively.

To explore the differential regulatory effects of LiBBX16p and LiBBX16g on anthocyanin-related promoters, dual-luciferase assays were performed by transient coinfiltration of *LiBBX16p/g* and promoters in *N. benthamiana* leaves (Fig. 7a). Compared with the empty vector, both LiBBX16p and LiBBX16g significantly activated the *LiCYP73A4*, *LiCYP73A13*, and *LiCHI* promoters. However, *LiBBX16p* induced ~1.8- to 4.8-fold higher luciferase activity on these promoters than *LiBBX16g*. No significant difference was observed between LiBBX16p and LiBBX16g in their activation of the *LiF3H* promoter. Notably, LiBBX16p, but not LiBBX16g, significantly activated the promoters of *LiF3GT* and *LiMYB5*, inducing ~3.7- and 5.3-fold increases in luciferase activity, respectively, compared with the control. Consistent with previous findings that *LiMYB75* expression correlates with anthocyanin accumulation in the leaves of *L. indica* [50], LiBBX16p activated the *LiMYB75* promoter ~2.2-fold higher than LiBBX16g (Fig. 7b). However, unlike the *in vivo* activation results observed in the dual-luciferase assay, electrophoretic mobility shift assay (EMSA) results showed that LiBBX16g, but not LiBBX16p, could directly bind to the promoter fragments of *LiF3GT* and *LiMYB75 in vitro* (Fig. 7c).

**Figure 8:**
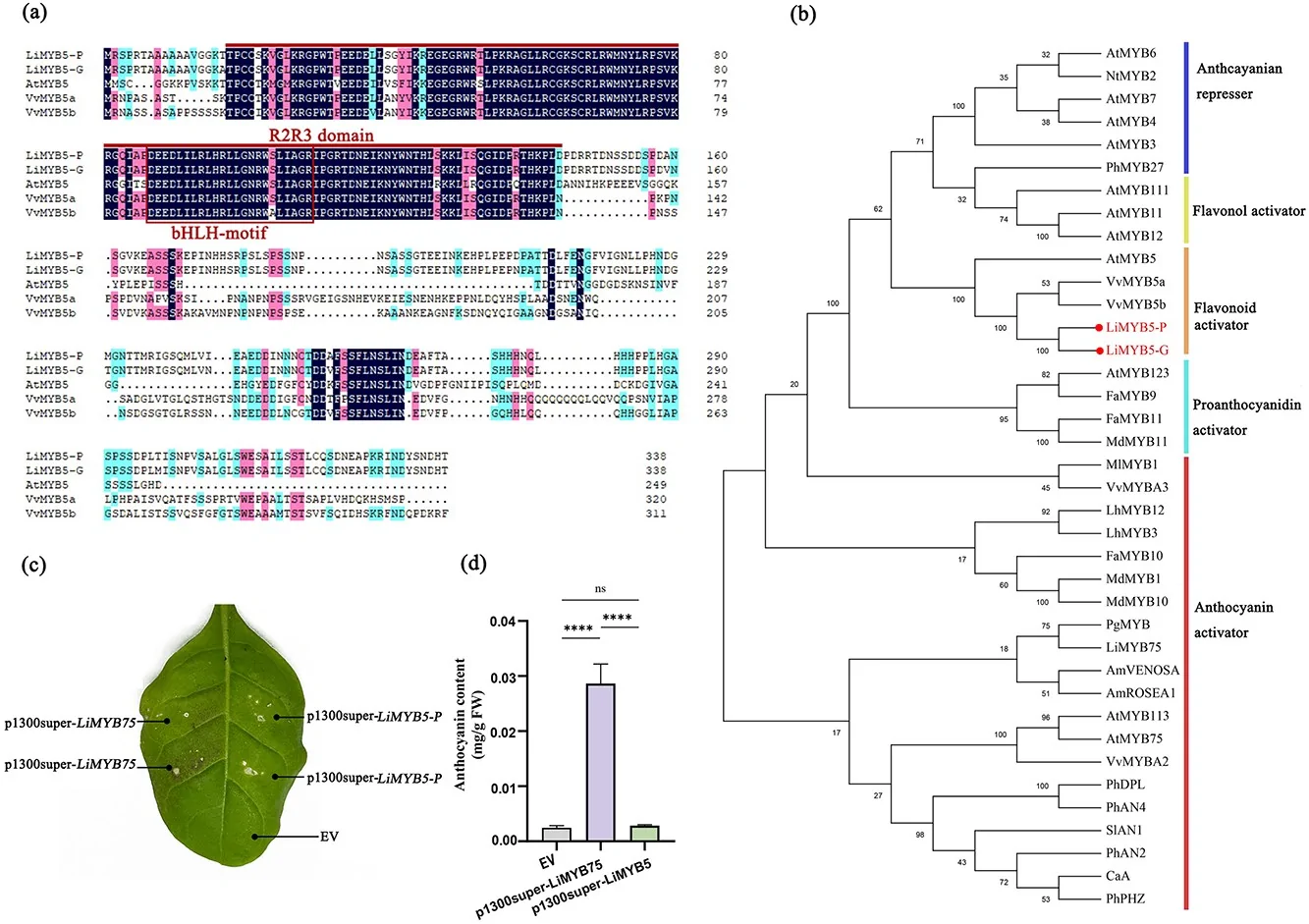
Amino acid sequence analysis, phylogenetic analysis, and functional verification of *LiMYB5*. (a) Alignment of amino acid sequences of LiMYB5 protein and three other species. (b) Phylogenetic tree of MYB proteins in different plant species. Plant species and GenBank accession numbers are listed in Table S14. (c) Leaf phenotypes of tobacco leaves transiently overexpressing *LiMYB75* and *LiMYB5-P* on the sixth day after transformation. EV represents empty vector transient transformation as a control; p1300super-*LiMYB75* represents transient transformation with *LiMYB75*, and p1300super-*LiMYB5-P* represents transient transformation with *LiMYB5-P*. (d) Total anthocyanin content in transiently transformed tobacco leaves. Asterisks represent significant differences by Student’s *t-*test (^****^P < 0.0001, ns means no significant difference).

**Figure 9:**
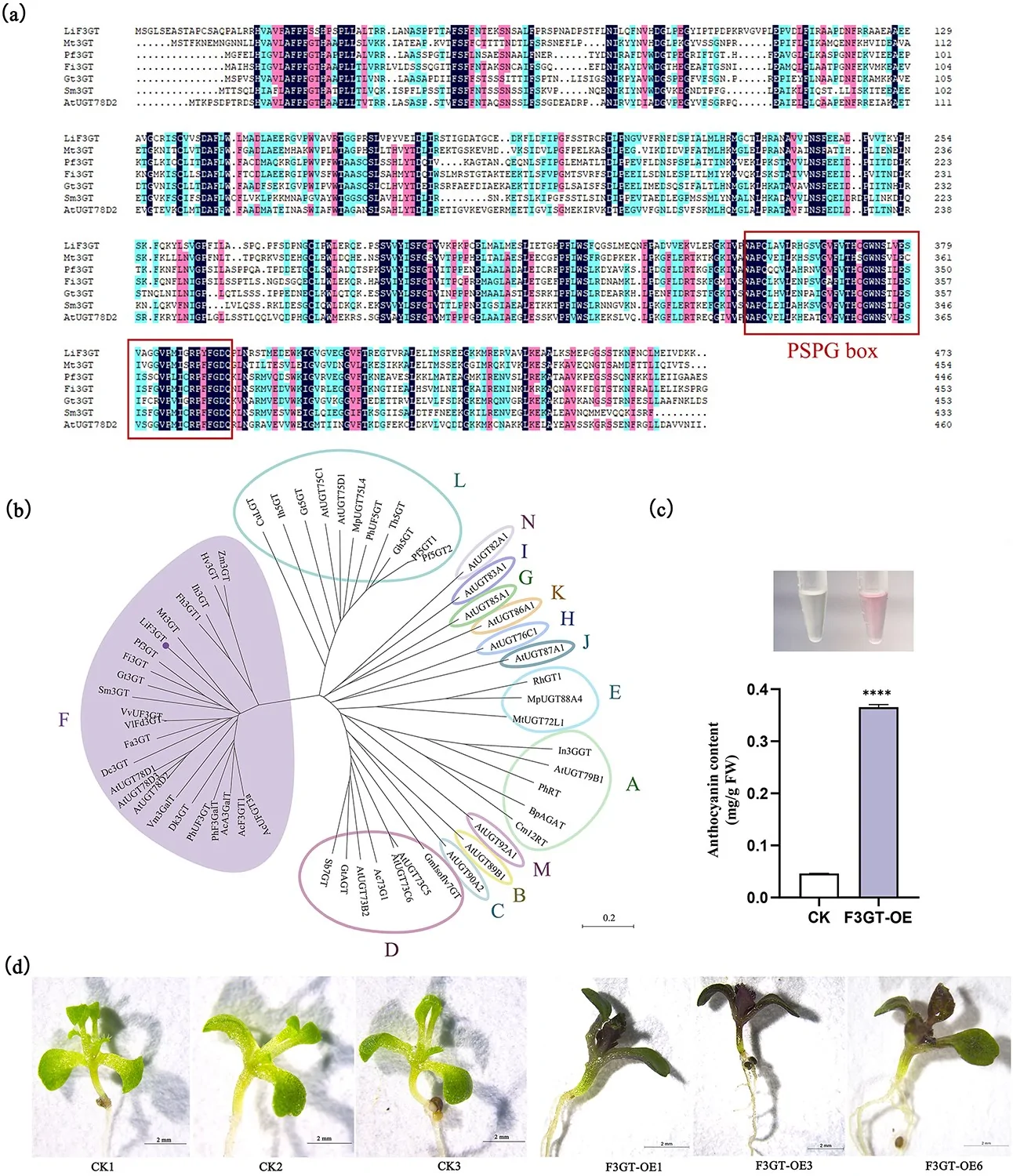
Amino acid sequence analysis, phylogenetic analysis, and functional verification of *LiF3GT*. (a) Alignment of amino acid sequences of LiF3GT protein and six other species. PSPG stands for Plant Secondary Product Glycosyltransferase. (b) Phylogenetic tree of UDP-glycosyltransferase (UGT) proteins in different plant species. Plant species and GenBank accession numbers are listed in Table S15. Fourteen groups (A–N) defined by Bowles *et al.* [53] are indicated. (c) Total anthocyanin contents of *Arabidopsis* seedlings transformed respectively by empty vector and *LiF3GT*. CK is the seedlings overexpressing empty vector of pSuper1300, and F3GT-OE is the seedlings overexpressing *LiF3GT*. (d) The phenotypes of 10-day-old transgenic *Arabidopsis* seedlings from the T2 generation of *LiF3GT*.

Based on LiBBX16p’s specific activation of *LiF3GT* and *LiMYB5 in vivo*, we further characterized these two candidate targets. Sequence analysis revealed that *LiMYB5* from purple- and green-leafed progeny (designated *LiMYB5-P* and *LiMYB5-G*) are both 1020 bp (Fig. S11), encoding 339-amino acid proteins that differ at seven residues. LiMYB5 contains a conserved R2R3 domain and a bHLH-binding motif at its N-terminus (Fig. 8a) and clusters with flavonoid-related activating MYB TFs, showing closest homology to VvMYB5a/b (Fig. 8b). The CDS of *LiF3GT* was identical between purple- and green-leafed progeny, with a length of 1422 bp (Fig. S11), encoding a 474-amino acid protein containing the conserved plant secondary product glycosyltransferase (PSPG) motif (Fig. 9a). LiF3GT clusters with the members of UDP-glycosyltransferase (UGT) family Group F in *Arabidopsis* (Fig. 9b), which are known to directly participate in anthocyanin biosynthesis [51, 52].

We conducted functional tests on these two genes. Transient overexpression in tobacco leaves showed that *LiMYB75* strongly promoted local pigment deposition and increased anthocyanin content, whereas *LiMYB5-P* overexpression had no such effect (Fig. 8c and d). Stable transformation of *LiF3GT* in *Arabidopsis* resulted in visibly purple leaves (Fig. 9d) and ~7.9-fold higher anthocyanin content than in controls (Fig. 9c).

Overall, in purple-leafed crape myrtle, the 32-bp deletion enhances LiBBX16’s activation of anthocyanin synthesis-related genes, particularly *LiF3GT* and *LiMYB75*. LiF3GT directly contributes to anthocyanin accumulation, whereas LiMYB75 upregulates the key structural genes *LiCHS* and *LiANS* [50], thereby promoting pigmentation. Thus, *LiF3GT* and *LiMYB75* are two key functional targets downstream of LiBBX16p.

## Discussion

### Application of *LiBBX16* deletion marker in breeding and genetic analysis of dominant homozygous lethal mutants

Molecular marker technology, as a cutting-edge breeding approach, offers several advantages including rapid and straightforward implementation, operational accuracy, ease of dissemination, and safety. This technology significantly enhances the precision of breeding objectives and increases the frequency of gene mutations, and greatly shortening the duration of the breeding process. In *Cucumis melo*, a KASP marker cosegregated with pericarp color phenotype was developed based on the SNP.G614331A mutation site of *CmAPRR2* gene [54]. In tea trees, a molecular marker derived from the insertion of a 181-bp transposon (purpleTE) in the promoter region of *CsMYB75*, and the marker can effectively distinguish purple-leafed progenies from green-leafed progenies [37]. In *P. mume*, a 1.8-Mb INV was identified from the haplotype C in ‘Meiren’ derived from ‘Pissardii’ and co-isolated with the purple leaf trait [40]. In our research, we developed an InDel marker from a 32-bp deletion in *LiBBX16* that can be used as a marker for identifying purple-leafed crape myrtle. This marker can accurately distinguish the perennial purple-leafed progenies, progenies with red young leaves and green mature leaves, and the green-leafed progenies at the seedling stage, which is a powerful tool for the molecular improvement of leaf color.

Interestingly, we found a type of extremely dwarf and embryonically lethal individuals called ‘mini violet’, and the electrophoresis results of all progenies of ‘mini violet’ contained only a single band at 192 bp (Fig. 3b). Subsequently, using the genomic DNA from the ‘mini violet’ individuals as a template, we cloned the full-length LiBBX16 and sequenced eight positive clones. The results indicated that all the LiBBX16 variants contained the same 32-bp deletion (Fig. S12), confirming that this genotype was homozygous for LiBBX16p. In Populations II and V, we found the segregation ratios of purple-leafed progenies to green leafed progenies was 37:18 and 100:60, excluding transiently surviving ‘mini violet’ individuals, respectively. A chi-square test revealed that both ratios conformed to the expected Mendelian ratio of 2:1 (*P* < 0.05), which aligns with the genetic pattern indicative of dominant homozygous lethality. Therefore, we propose that ‘mini violet’ is a homozygous dominant individual, the purple-leafed plant is a heterozygous dominant individual, and the green-leafed plant is a homozygous recessive individual in *L. indica*. It is speculated that the segregation ratio of these three phenotypes conforms to 1:2:1, which is consistent with Mendelian genetic inheritance. Several plant species have been identified with lethal mutations that lead to embryo or seedling death, including the seedling lethality and chlorophyll deficiency due to the ATYCO/RP8/CDB1 mutations in *Arabidopsis* [55], the albino lethal phenotype at the seedling stage due to ALS1 functional deficiency in rice [56], and programmed cell death observed in rice *Osspl26* dominant homozygous mutants caused by excessive accumulation of H_2_O_2_ [57]. The mechanisms underlying lethality in plants are typically associated with disruptions of developmental programs or metabolic homeostasis collapse. These disruptions manifest as disturbances in photosynthetic pigment metabolic pathways, abnormal developmental processes (such as meristem defects) and excessive accumulation of toxic substances like anthocyanins [58–60]. Although further experimental verification is needed to elucidate the mechanism behind the homozygous lethality of LiBBX16p mutant in *L. indica*, this mutant offers unique genetic material for analyzing regulatory networks involved in plant growth and development as well as cell death signaling pathways.

### *LiBBX16p* is an important transcription factor for anthocyanin synthesis in purple-leafed crape myrtle

Previous studies have demonstrated that several BBX TFs are involved in the regulation of anthocyanin biosynthesis [11, 61, 62]. For example, in *Arabidopsis*, BBX20/21/22 interact with HY5 to enhance anthocyanin accumulation by transcriptionally activating genes involved in anthocyanin biosynthesis while concurrently inhibiting hypocotyl growth [24, 63, 64]. In contrast, BBX24/25 directly interact with both HY5 and COP1, thereby suppressing HY5’s activation on downstream target genes CHS and CHI and enhancing the function of COP1. This interaction ultimately leads to the repression of both anthocyanin accumulation and hypocotyl growth [65–67]. Homologs have since been characterized in other species, exhibiting significant functional divergence, for instance, *MdCOL4* inhibits anthocyanin accumulation in apple peel [68], while *LvBBX24* promotes it in lily petals [69]. The results indicated that despite their sequence homology, the roles of BBX24 orthologs in regulating anthocyanin biosynthesis vary markedly across different plant species. In *L. indica*, Deng *et al.* [70] successfully cloned the *LiBBX4* gene, and found that the gene is homologous to *AtBBX24* of *Arabidopsis thaliana* and is located on chromosome 3, where it functions as a negative regulator of anthocyanin accumulation. The *LiBBX16* identified in our study resides on chromosome 12 and exhibits homology to both AtBBX24 and AtBBX25 of *A. thaliana*. Subsequent experiments utilizing VIGS in *L. indica* and stable transformation in *A. thaliana* demonstrated that *LiBBX16p* functions as a positive regulator of anthocyanin biosynthesis (Fig. 5), which is consistent with previous research results on *Pyrus pyrifolia* [47, 71, 72] and *Gossypium hirsutum* [46]. In the studies of anthocyanin accumulation in *P. pyrifolia*, it was found that PyBBX24^δN14^ directly activates the transcription of *PyUFGT* and *PyMYB10* through interaction with PyHY5, while the full-length form of PyBBX24 does not possess this capability [47, 71]. Moreover, it was found that Ppbbx24-del can also directly activate the transcription of *PpCHS* and *PpCHI* to regulate anthocyanin accumulation*,* whereas PpBBX24 does not exhibit this capability [72]. In our study, we first demonstrated that allelic variation in LiBBX16 can differentially regulate multiple tiers of anthocyanin synthesis, spanning core structural genes in the phenylpropanoid (*LiCYP73A4*, *LiCYP73A13*) and flavonoid pathways (*LiCHI*, *LiF3GT*) to upstream regulators (*LiMYB75*, *LiMYB5*), through Dap-seq and dual-luciferase assays (Figs S10 and 7b).

Furthermore, our study validated the function of *LiF3GT*, a downstream target gene of LiBBX16p, and confirmed its role in influencing anthocyanin accumulation (Fig. 8c). *LiF3GT* belongs to the F group of the UGT family in *A. thaliana*. Members of this group have been reported to participate in the glycosylation modification process of anthocyanins. Among them *AtUGT78D2* has been shown to catalyze the glycosylation of cyanidin aglycone, and the main product is cyanidin-3-*O*-glucoside [73]. However, the main product of *LiF3GT* catalyzing the glycosylation modification of anthocyanins needs further experiments. Additionally, *LiMYB5* is another important downstream target gene of LiBBX16p, and it is classified as a transcription factor that activates flavonoid synthesis and may influence the final levels of anthocyanin by regulating the accumulation of total flavonoids, the precursors for anthocyanins biosynthesis [74, 75]. Although the specific roles of the downstream target genes of LiBBX16p in anthocyanin synthesis remains to be further analyzed, our findings have revealed a novel pathway for anthocyanin biosynthesis mediated by the *BBX24/25* gene via a transcriptional regulatory network, laying a foundation for further elucidation of its molecular mechanisms.

### The 32-bp deletion in *LiBBX16* alters protein structure and its biological function

In many plant species, BBX proteins typically contain not only conserved B-box domain at their N-terminal region but also NLS and VP motifs in their C-terminal region [65]. Functional diversification among BBX proteins is often driven by C-terminal variations, as illustrated in *Arabidopsis* where the distinct photomorphogenic roles of BBX24 and BBX21 are determined by their respective C-termini, while their N-terminal B-box domains remain interchangeable [76]. In this study, we found that LiBBX16a, LiBBX16g, and LiBBX16p each possess two N-terminal B-box domains, but differ in their C-terminal regions: LiBBX16a and LiBBX16g contain NLS and VP motifs, whereas LiBBX16p lacks both due to the 32-bp deletion. This deletion alters the subcellular localization of LiBBX16p, partially impairing its nuclear entry (Fig. 4f). In eukaryotes, loss of targeting signals such as the NLS often disrupts protein function [77, 78]. For instance, the NLS of AtBBX24 in *Arabidopsis* has been shown to play a critical role in its function within the light signaling pathway [79]. Similarly, the VP motif serves as a binding site for the E3 ubiquitin ligase COP1 [80]. In pear, the VP-deleted variant PpBBX24-del escapes COP1-mediated degradation, leading to increased stability and enhanced anthocyanin accumulation [81].

In addition to these possible effects on subcellular localization and protein stability, the 32-bp deletion also directly modulates the intrinsic transcriptional activity of LiBBX16. Transcriptional activity analysis revealed that the 32-bp deletion creates a novel transcriptional activation domain (AD) in the C-terminus of LiBBX16p—a domain absent in LiBBX16g (Fig. S8). This structural alteration provides a direct molecular basis for the significantly enhanced transcriptional activation capacity of LiBBX16p. Although the isolated C-terminal fragment exhibited strong self-activation in yeast, the full-length LiBBX16p protein did not, suggesting that in the native protein context the nascent AD may be partially masked or may require plant-specific interactors or modifications for full activity.

Consistent with the hypothesis that LiBBX16p acts through a distinct mechanism from LiBBX16g, an interesting discrepancy was observed between the *in vivo* and *in vitro* binding assays. While LiBBX16p significantly activated the promoters of *LiF3GT* and *LiMYB75* in dual-luciferase assays, it failed to bind directly to these promoters in EMSA. In contrast, LiBBX16g retained the ability to directly interact with these promoters, suggesting that the transcriptional activation mediated by LiBBX16p may not require direct DNA binding. We hypothesized that LiBBX16p functions as a coactivator that is recruited to target promoters through protein–protein interactions with other DNA-binding TFs *in vivo*, such as members of the HY5 [22, 23, 66], or MYB families [82].

Based on the classic COP1-HY5-BBX regulatory module [22, 66, 79] and the above discussion, we propose the following nuclear import mechanism to explain how the 32-bp deletion in *LiBBX16p* may drive the purple leaf trait in crape myrtle. The truncation retains LiBBX16p partially in the cytoplasm, potentially allowing it to escape COP1-mediated degradation and accumulate. Under light conditions, cytoplasmic LiBBX16p may be imported into the nucleus via interaction with stably nuclear-localized TFs or other protein partners—a ‘piggy-back’ mechanism common among proteins lacking a canonical NLS [83]. This mechanism not only compensates for the missing NLS but also facilitates the recruitment of LiBBX16p to target promoters as a coactivator. Once inside the nucleus, consistent with its inability to directly bind DNA (Fig. 7c), LiBBX16p likely forms a stable and highly active transcriptional complex with partner TFs, leading to persistent activation of key anthocyanin biosynthesis genes and ultimately resulting in the purple-leaf phenotype. This mechanism provides a novel molecular perspective on how a single structural variation rewires protein function to generate leaf color diversity, although further validation through experiments such as light-dependent subcellular localization assays, interaction-dependent nuclear import assays, and protein half-life measurements is required.

## Conclusion

**Figure 10:**
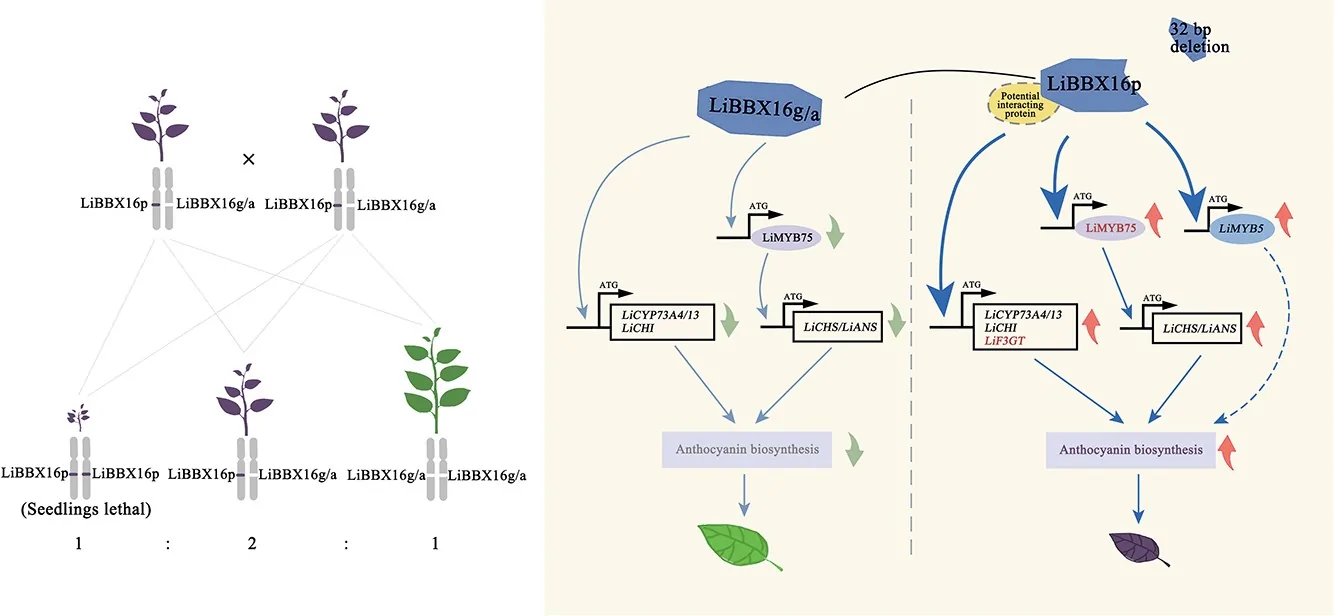
Genetic (left) and working (right) model of *LiBBX16* in regulating the purple leaf trait in *L. indica*. (Left) Crosses between purple-leafed and purple-leafed individuals of *L. indica* produced progenies with a segregation ratio of 1 mini violet: 2 purple leaf: 1 green leaf. (Right) In the nucleus, LiBBX16p may be recruited to the promoters of *LiCYP73A4*/*13*, *LiCHI*, *LiF3GT*, *LiMYB75*, and *LiMYB5* through protein–protein interactions, functioning as a transcriptional coactivator. This recruitment ultimately leads to anthocyanin accumulation and the development of the purple leaf trait. Arrow thickness signifies activation strength; solid arrows indicate verified pathways, and dashed arrows indicate proposed pathways. Gene names highlighted in red denote those specifically activated by LiBBX16p.

In summary, for the first time, we clarified through forward genetics that the purple leaf trait of *L. indica* is a dominant trait controlled by a single gene. The key candidate gene, *LiBBX16*, was successfully identified, and a 32-bp deletion within its coding sequence was found to cosegregate with the purple leaf trait. LiBBX16p, the allele responsible for the purple leaf trait, which carries the 32-bp deletion, significantly activates the promoters of anthocyanin-related genes (*LiF3GT* and *LiMYB75*) *in vivo* but fails to bind these promoters directly *in vitro.* In contrast, LiBBX16g retains direct DNA-binding capability while exhibiting weaker transcriptional activation. The 32-bp deletion in *LiBBX16p* removes the C-terminal NLS and VP motifs, generates a novel transcriptional activation domain, and causes the protein to be partially retained in the cytoplasm. Furthermore, all plants homozygous for *LiBBX16p* are seedling lethal, indicating an additional role for this gene in growth and development. These findings provide direct evidence for the molecular mechanism by which allelic variation in *LiBBX16* regulates anthocyanin biosynthesis and offer a potential target gene for leaf color improvement in ornamental plants (Fig. 10).

## Materials and methods

### Hybrid population construction and phenotypic observation

**Figure 11:**
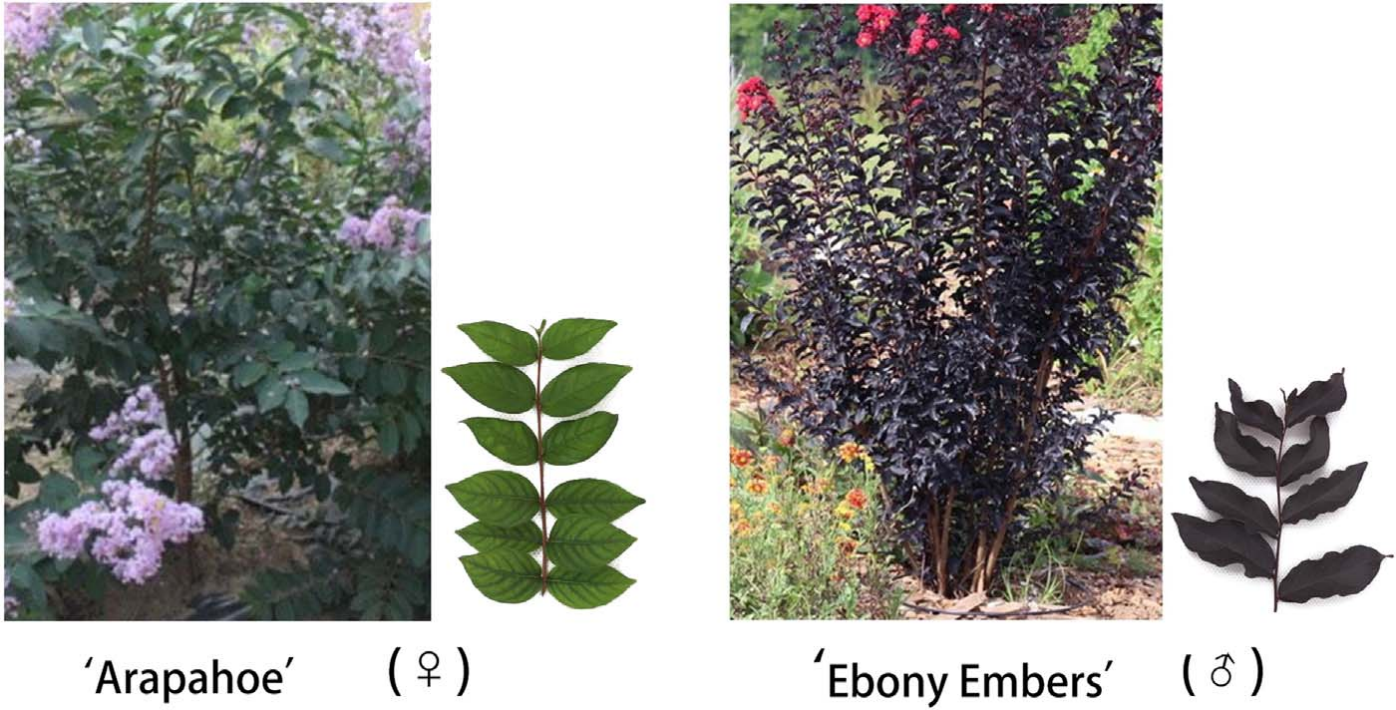
Leaf color of hybrid parents of Population I (the left is the female parent ‘Arapahoe’, and the right is male parent ‘Ebony Ember’).

In the summer of 2017, the female parent ‘Arapahoe’ (green leaves) and the pollen donor ‘Ebony Embers’ (purple leaves) (Fig. 11) were crossed to construct Population I [50]. In the summer of 2022, the individuals with extreme purple and green leaves in Population I were selected for hybridization, and four hybrid populations were established (Fig. S1), including Population II: the progeny No. 23 (purple leaves) × No. 29 (purple leaves); Population III: No. 80 (green leaves) × No. 29; Population IV: No. 80× No. 37 (green leaves). In the summer of 2023, population of ‘Ebony Embers’ × No. 32 (purple leaves) in Population I was constructed as Population V (Fig. S1). The hybrid seeds obtained were sown in pots (25 × 25 cm) in the greenhouse of the Plant Science Center of Beijing Forestry University (40°0′35″N, 116°20′40″E, and 51 m above sea level) in March of the next year after obtaining the seeds.

From June to July in 2023 and 2024, respectively, the mature leaves on the 1-year-old shoots were observed by visual method under natural light conditions. Phenotypes were classified in a binary manner: individuals with any purple pigmentation were categorized as purple, and those without as green. The leaf color data were processed using Excel and the genetic patterns of purple leaf trait were analyzed by χ^2^ test.

### Anthocyanin content determination

Anthocyanin was extracted from 0.1 g fresh mature leaves of each progeny in Population I and then mixed with 25 ml 1% HCl–methanol solution, respectively, and incubated in dark for 12 h. The absorbance of the solution was determined at 532 and 650 nm using BioMate 3S UV spectrophotometer (Thermo Scientific, USA), the pigment content was calculated as described previously [84].

### BSA sequencing and analysis

‘Arapahoe’ and ‘Ebony Embers’ were used to construct two parent pools, respectively. Twenty-one progenies with extremely purple leaves (anthocyanin content in leaves is >4.2 mg/g) and extremely green leaves (anthocyanin content in leaves is <0.5 mg/g (Table S16)) in Population I were selected respectively to construct the purple-leafed pool and green-leafed pool. An equal amount of DNA sample was collected from each individual. Genomic DNA of young leaves was isolated using New Type Plant DNA Kit (DN15, Aidlab, Beijing, China). The extracted DNA samples were tested for quantity and quality using a Nanodrop 2000 Spectrophotometer (Thermo Scientific, USA). The parents and pooled DNAs were sequenced on an Illumina Hiseq ×10 PE150 (Genedenovo Biotechnology Co., Ltd, Guangzhou, China).

The raw reads were first filtered to obtain high-quality clean reads. The clean reads were aligned to the *L. indica* reference genome (BioProject ID: PRJNA1058153) using Burrows–Wheeler Aligner [85, 86], and the results were sorted and tagged with duplicated sequences using Picard (http://sourceforge.net/projects/picard/). Coverage statistics were performed using bedtools (v2.27.1) [85]. The processed alignment files were used for variant calling, which was performed on each sample using GATK’s Variant Filtration with appropriate parameter settings. SNPs and InDels were annotated by ANNOVAR software [87]. The Δ(SNP/InDel-index), the Euclidean distance calculation and G statistic value were used for mapping candidate regions associated with leaf color phenotype [88, 89].

### Cloning and genotyping analysis of *LiLDOX*

The CDS of *LiLDOX* was cloned from cDNA obtained by reverse transcription of RNA extracted from pooled leaf samples (10 purple- and 10 green-leafed progenies in Population I). The promoter region (a 1806-bp fragment upstream of ATG) was cloned from genomic DNA extracted from the same set of pooled leaf samples (primers listed in Table S17). Subsequently, the CDS of *LiLDOX* of nine individual purple- and green-leafed progenies was cloned, sequenced, and analyzed separately to identify mutations and determine genotypes. Total RNA extraction and cDNA synthesis followed a previously described method [50].

### Identification of *LiBBX16* genotype

To verify the association between the selected InDel and the trait of purple leaves, we designed a pair of primers that encompasses the deleted region (*LiBBX16*-check-F: 5′- GAGTTCGGGGATCTAGACTGGC-3′ and *LiBBX16*-check-R: 5′- AAGTGCTC CTCATCATCGTCGT-3′) according to the sequence information. PCR amplification was then performed using this pair of primers on the DNA samples of 40 purple-leafed progenies and 39 green-leafed progenies in Population I, 9 progenies with purple leaves and extremely short plant type in Population V, 3 purple-leafed progenies and 3 green-leafed progenies in Population V, and 13 cultivars with different leaf color (Table S9). PCR amplification and agarose gel electrophoresis were performed as described previously [90].

### Cloning and bioinformatics analysis of *LiBBX16*

The fragments of genomic DNA and promoters (a 1907-bp fragment upstream of ATG) of *LiBBX16* were cloned from the purple- and green-leafed pools of Population I, as well as from randomly selected pools of 10 purple- and green-leafed progenies. The CDS of *LiBBX16* was cloned from the purple- and green-leafed progenies in Population I (primers used are listed in Table S17). The CDS of *LiBBX16* existed only in the purple-leafed progenies was named *LiBBX16p*, the CDS of *LiBBX16* that existed only in the green-leafed progenies was named *LiBBX16g*, and the CDS of *LiBBX16* that existed in both purple- and green-leafed progenies was named *LiBBX16a*. The extraction methods of total RNA and cDNA were as described previously [50].

The hidden Markov model (HMM) for the conserved B-box domain (Pfam ID: PFPF00643) was retrieved from the Pfam protein database (https://pfam.xfam.org/). Candidate BBX proteins from the *L. indica* reference genome were identified using HMMER software when the E-value threshold is <1e-5 and a score is >90. Subsequently, the results were analyzed by TBtools software 2.310 for intraspecific collinearity. The protein sequences of BBX family in *A. thaliana* were downloaded from the TAIR database (https://www.arabidopsis.org/). The amino acid sequences of BBX in the genome of *L. indica* and other species were aligned using the Muscle tool of MEGA 10. 0. 5. The phylogenetic tree was constructed by using the neighbor-joining method in phylogeny tool, and 2000 bootstrap replicates were set to assess the reliability of the phylogenetic tree. The amino acid sequence of the candidate gene branch was submitted to TBtools software for sequence similarity analysis, and then the branch sequence was submitted to MEME Suite (https://meme-suite.org/meme/tools/meme) for motif analysis and plotted using TBtools software 2.310.

### Subcellular localization

*35S::LiBBX16a-GFP*, *35S::LiBBX16p-GFP*, and *35S::LiBBX16g-GFP* constructs were generated by inserting the CDSs of *LiBBX16a, LiBBX16p*, and *LiBBX16g* into the pCAMBIA1300 super vector through *Xba I* and *Kpn I* restriction sites, respectively (primers used are listed in Table S17). Subcellular localization was performed according to a method described previously [91].

### Transcriptional activation activity assay

To further investigate the functional properties of LiBBX16, the following constructs were generated in the pGBKT7 (BD) vector as baits: the N-terminal region (amino acids 1–99), the C-terminal region of LiBBX16p (amino acids 100–221), and LiBBX16g (amino acids 100–249), as well as the full-length sequences of LiBBX16p and LiBBX16g (primers are listed in Table S17). Each bait construct was cotransformed with the empty pGADT7 (AD) vector into the yeast strain Y2H Gold (YC1002S, WEIDI, China) to assess self-activation activity. AD-T + BD-Lam and AD + BD served as negative controls, while AD-T + BD-53 served as the positive control. Yeast transformation and autoactivation assays were performed as described by Feng *et al*. [50]

### Virus-induced gene silencing assay on purple-leaf crape myrtle

A gene-specific fragment of *LiBBX16p* (228 bp in length) was inserted into the *EcoR I* - *BmaH I* site of pTRV2 vector to produce pTRV2-*LiBBX16p* construct (primers used are listed in Table S17). One-year-old rooted cuttings of the progeny No. 114 with purple leaves in Population I were used as materials. Prune the plants and conduct experiments when the new shoots grow to have two to three internodes. The VIGS experiments were conducted as previously specified [50]. The newly formed young leaves 30 days after silencing by VIGS were collected for qRT-PCR analysis. Primers used are listed in Table S17.

### Transient color assays in tobacco leaves

The CDSs of *LiMYB5-P* and *LiMYB75* were isolated and inserted into the pCAMBIA1300 super vector (primers used are listed in Table S17). *Agrobacterium*-mediated transient transformation was performed according to a method described previously [50]. The pCAMBIA1300 super vector (EV) was used as a negative control. At 5 days after infiltration, the leaves with injections were collected for phenotypic assessment, anthocyanin quantification, and transcript abundance analysis.

### *Agrobacterium*-mediated stable transformation in *Arabidopsis*

The pCAMBIA1300super, pCAMBIA1300super-*35S::LiBBX16a-GFP*, pCAMBIA1300super-*35S::LiBBX16p-GFP*, pCAMBIA 1300super-*35S:: LiBBX16g-GFP*, and pCAMBIA1300super-*35S::LiF3GT-GFP* vectors were used for genetic transformation of *A. thaliana* by floral dip method [92]. The T1 and T2 generation plants were verified by PCR, and the seeds of the positive lines were sown on MS medium with hygromycin resistance. Two weeks later, the seedlings were transplanted to pots and grown in a glasshouse. Anthocyanin content and the transcript levels of *LiBBX24a, LiBBX24p*, *LiBBX24g*, and *LiF3GT* were measured using 10-day-old seedlings grown on MS medium.

### DNA affinity purification sequencing

The genomic DNA was extracted from the leaves of 1-year-old rooted cuttings of the progeny No. 114 with purple leaves in Population I using the CTAB method to prepare the DNA library. The CDS of *LiBBX16p* was inserted into the HaloTag expression vector to generate recombinants. A mixture of the Magne HaloTag Beads and Halo-*LiBBX16p* was incubated with the DNA library. The DNA fragment bound to the *LiBBX16p* was eluted from the beads and sequenced on the Illumina NovaSeq 6000 platform. The clean reads were aligned to the *L. indica* reference genome using BWA-MEM [93]. The sequenced data were identified for peaks using MACS [94]. We employed MEME-ChIP to identify the core motifs [95]. DNA affinity purification sequencing (DAP-seq) was performed according to the method described previously [96, 97].

### Dual-luciferase reporter assay

The promoter regions of *LiCYP73A4* (20–1353 bp upstream of ATG), *LiCYP73A13* (536–1492 bp upstream), *LiCHI* (2364 bp upstream), *LiF3H* (2023 bp upstream), *LiF3GT* (1760 bp upstream), *LiMYB5* (1049 bp upstream), and *LiMYB75* (2008 bp upstream) were amplified from genomic DNA of ‘Ebony Embers’ and purple-leafed individuals in population I using specific primers. All cloned promoter fragments encompass the DAP-seq peaks identified for LiBBX16p. These fragments were inserted into the pGreenII 0800-LUC vector through *Kpn I* and *BmaH I* restriction sites (primers used are listed in Table S17). The full-length CDSs of *LiBBX16p* and *LiBBX16g* were cloned into the pGreen II 62-SK vector through *Xba I* and *Kpn I* sites (primers used are listed in Table S17). Dual-luciferase assays were performed as previously described [50]. After 3 days of infiltration, firefly (LUC) and *Renilla* (REN) luciferase activities were measured using the dual-luciferase reporter assay reagents (DL101-01, Vazyme, China) and an enzyme calibration (Envision, Revvity, UK). The ratios of LUC and REN were expressed as activation or repression.

### Electrophoretic mobility shift assay

A5′-biotinylated oligonucleotide (5′-ATG CAT GCA TGC ATG CAT GCA TGC-3′) was used as the probes. The probes were incubated with the nuclear extract at room temperature for 30 min. The entire reaction mixture was run on a nondenaturing 0.5× TBE 6% polyacrylamide gel for 1 h at 60 V at 4°C and then transferred onto Biodyne® B nylon membranes (Pall Corporation). Signals were visualized with reagents included in the kit and ChemiDoc XRS (Bio-Rad Laboratories, UAS).

### Statistical analysis

The data were analyzed using Student’s *t*-test and one-way analysis of variance combined with Tukey’s *hoc* test of SPSS 25.0. Data analysis was visualized and utilized for GraphPad Prism 8.0.2 (San Diego, California, USA).

## Supplementary Material

Web_Material_uhag179

## Data Availability

The raw sequence data of BSA-seq is available in the National Genomics Data Center (NGDC) with the accession number PRJCA046681. The DAP-seq data have been deposited to the NGDC with the accession number PRJCA046707. The GenBank accession numbers for the genes are LC897221 (LiBBX16a), LC897222 (LiBBX16g), LC897223 (LiBBX16p), and LC897224 (LiF3GT).
