# Supplementary material for: A 32-bp deletion in LiBBX16 underlies the dominant purple leaf trait in crape myrtle
Source: Hortic Res. 2026 Apr 29;13(10):uhag179. doi: 10.1093/hr/uhag179 (PMC13600421; doi:10.1093/hr/uhag179)
Supplement: Supplementary file 1 [file web_material_uhag179.zip › Revised-Supplementary Figures.docx]

Figure S1 Leaf color of some hybrid progenies of Population I. (The lower left corner is the number of the plant in Population I).

Figure S2 Alignment of the amino acid sequence of LiLDOXa, LiLDOXg and LiLDOXp. (LiLDOXa denotes the sequence present in both purple- and green-leafed progenies; LiLDOXg and LiLDOXp refer to sequences exclusive to green-leafed and purple-leafed progenies, respectively.)

Figure S3 Analysis of promoters of *LiLDOX* in crape myrtle. (a) Alignment of the promoter sequences of *LiLDOX* in purple- and green leaved crape myrtle. (b) Visual analysis of cis-acting elements of promoter of *LiLDOX*.

Figure S4 Analysis of promoters of *LiBBX16* in crape myrtle. (a) Alignment of the promoter sequences of *LiBBX16* in purple- and green leaved crape myrtle. (b) Visual analysis of cis-acting elements of promoter of *LiBBX16*.

Figure S5 Primer locations at the upstream and downstream positions of the deletion base of *LiBBX16* in crape myrtle (The letters F and R represent the forward and reverse primers, respectively).

Figure S6 Phenotypic characteristics of “mini violet”, purple- and green-leafed progenies in Population V. (a) The progenies in Population V three months after sowing. (b) The progenies in Population V four months after sowing. From left to right were “mini violet”, purple- and green-leafed plants.

Figure S7 Chromosome location of *LiBBXs* in *L. indica*.

Figure S8 Detection of self-activation of LiBBX16 and its variants in yeast two-hybrid system. (a) Schematic diagram of the LiBBX16 protein and its variants. (b) Interactions between LiBBX16p, LiBBX16p-C, LiBBX16g, LiBBX16g-C, LiBBX16-N and AD determined by a yeast two-hybrid assay.

Figure S9 Venn diagram of candidate genes in two comparisons of promoter-associated genes by DAP-seq and differential genes by RNA-seq. A represents the related gene set of peaks annotated as promoters in DAP-seq; B represents the differential gene set in RNA-seq.

Figure S10 DAP-seq binding peaks of *LiCYP73A4*, *LiCYP73A13*, *LiCHI*, *LiF3GT*, *LiMYB5* and *LiMYB75*.

Figure S11 Alignment of coding sequences of *LiMYB5* (a) and *LiF3GT* (b) in purple- and green-leafed progeny. (114P and 60G represent progeny No.114 (purple leaves) and No.60 (green leaves), respectively, from population I.)

Figure S12 Alignment of the full-length genomic DNA of *LiBBX16* in “mini violet” plant.
