# Supplementary material for: A 32-bp deletion in LiBBX16 underlies the dominant purple leaf trait in crape myrtle
Source: Hortic Res. 2026 Apr 29;13(10):uhag179. doi: 10.1093/hr/uhag179 (PMC13600421; doi:10.1093/hr/uhag179)
Supplement: Supplementary file 1 [file web_material_uhag179.zip › Revised-Supplementary Tables.docx]

Table S1 BSA sequencing data statistics for the ‘Arapahoe’×‘Ebony Embers’ population

| **Sample** | **Raw reads (bp)** | **Clean reads (bp)** | **GC rate**  **（%）** | **Q20 rate**  **（%）** | **Q30 rate**  **（%）** |
| --- | --- | --- | --- | --- | --- |
| BD4 | 12,612,521,400 | 12,278,446,629 | 41.50 | 97.34 | 93.12 |
| M54 | 13,591,428,000 | 13,306,302,950 | 40.87 | 95.99 | 89.96 |
| PL-F1 | 10,889,805,300 | 10,596,903,301 | 41.58 | 96.68 | 91.71 |
| GL-F1 | 11,377,505,400 | 11,131,200,318 | 41.59 | 94.76 | 87.02 |

Note: BD4: Abbreviation for ‘Ebony Embers’, which is the code for the pool of purple-leafed parent; M54: Abbreviation for ‘Arapahoe’, which is the code for the pool of green-leafed parent; PL-F1: the mixed pool of purple-leafed F_1_ progenies; GL-F_1_: the mixed pool of green-leafed F_1_ progenies.

Table S2 BSA sequencing data alignment results for the ‘Arapahoe’×‘Ebony Embers’ population

| **Sample** | **Average depth（×）** | **Total mapped**  **rate（%）** | **Cover 4×（%）** | **Cover 10×（%）** | **Cover 20×**  **（%）** |
| --- | --- | --- | --- | --- | --- |
| BD4 | 39 | 93.48 | 90.46 | 84.07 | 66.05 |
| M54 | 42 | 91.79 | 91.84 | 86.10 | 71.09 |
| PL-F1 | 34 | 92.70 | 92.43 | 82.37 | 57.43 |
| GL-F1 | 39 | 92.32 | 92.29 | 82.88 | 59.59 |

Note: BD4: Abbreviation for ‘Ebony Embers’, which is the code for the pool of purple-leafed parent; M54: Abbreviation for ‘Arapahoe’, which is the code for the pool of green-leafed parent; PL-F1: the mixed pool of purple-leafed F_1_ progenies; GL-F_1_: the mixed pool of green-leafed F_1_ progenies.

Table S3 SNP information statistics of BSA-Seq in ‘Arapahoe’×‘Ebony Embers’ population

| **Sample** | **SNP number** | **Ts** | **Tv** | **Ts/Tv** | | **Hom** | **Het** | **Heterozygo-sity rate (%)** |
| --- | --- | --- | --- | --- | --- | --- | --- | --- |
| BD4 | 2,864,299 | 1,837,385 | 1,026,914 | 1.79 | 1,195,465 | | 1,668,834 | 58.26 |
| M54 | 3,888,877 | 2,475,223 | 1,413,654 | 1.75 | 1,146,296 | | 2,742,581 | 70.52 |
| PL-F1 | 4,248,648 | 2,714,512 | 1,534,136 | 1.77 | 808,249 | | 3,440,399 | 80.98 |
| GL-F1 | 4,322,664 | 2,759,841 | 1,562,823 | 1.77 | 857,772 | | 3,464,892 | 80.16 |

Note: BD4: Abbreviation for ‘Ebony Embers’, which is the code for the pool of purple-leafed parent; M54: Abbreviation for ‘Arapahoe’, which is the code for the pool of green-leafed parent; PL-F1: the mixed pool of purple-leafed F_1_ progenies; GL-F_1_: the mixed pool of green-leafed F_1_ progenies.

Table S4 InDel information statistics of BSA-Seq in ‘Arapahoe’×‘Ebony Embers’ population

| **Sample** | **In/Del number** | **Hom** | **Het** | **Heterozygosity rate (%)** |
| --- | --- | --- | --- | --- |
| BD4 | 322,642 | 143,725 | 178,917 | 55.45 |
| M54 | 443,113 | 138,515 | 304,598 | 68.74 |
| PL-F1 | 474,019 | 96,262 | 377,757 | 79.69 |
| GL-F1 | 482,837 | 103,805 | 379,032 | 78.50 |

Note: BD4: Abbreviation for ‘Ebony Embers’, which is the code for the pool of purple-leafed parent; M54: Abbreviation for ‘Arapahoe’, which is the code for the pool of green-leafed parent; PL-F1: the mixed pool of purple-leafed F_1_ progenies; GL-F_1_: the mixed pool of green-leafed F_1_ progenies.

Table S5 Candidate intervals identified by the SNP-index method at the 99% confidence threshold in BSA analysis of ‘Arapahoe’×‘Ebony Embers’ population

| **Chromosome** | **Start** | **End** | **Candidate region（Mb）** |
| --- | --- | --- | --- |
| Chr8 | 5,930,001 | 7,270,000 | 1.34 |
| Chr12 | 8,450,001 | 11,890,000 | 3.44 |

Table S6 Candidate intervals identified by the ED method at the 99% confidence threshold in BSA analysis of ‘Arapahoe’×‘Ebony Embers’ population

| **Chromosome** | **Start** | **End** | **Candidate region（Mb）** |
| --- | --- | --- | --- |
| Chr8 | 6,290,001 | 7,210,000 | 0.92 |
| Chr12 | 440,001 | 1,070,000 | 0.63 |
| Chr12 | 8,480,001 | 11,920,000 | 3.44 |

Table S7 Candidate intervals identified by the G-statistic method at the 99% confidence threshold in BSA analysis of ‘Arapahoe’×‘Ebony Embers’ population

| **Chromosome** | **Start** | **End** | **Candidate region（Mb）** |
| --- | --- | --- | --- |
| Chr12 | 400,001 | 1,230,000 | 0.83 |
| Chr12 | 8,270,001 | 11,920,000 | 3.65 |
| Chr12 | 11,970,001 | 12,530,000 | 0.56 |

Table S8 Candidate intervals obtained in BSA analysis of ‘Arapahoe’×‘Ebony Embers’ population

| **Chromosome** | **Start** | **End** | **Candidate region（Mb）** |
| --- | --- | --- | --- |
| Chr8 | 5,930,001 | 7,270,000 | 1.34 |
| Chr12 | 400,001 | 1,230,000 | 0.83 |
| Chr12 | 8,270,001 | 11,920,000 | 3.65 |
| Chr12 | 11,970,001 | 12,530,000 | 0.56 |

Table S9 Leaf color phenotypes of cultivars used for the leaf color InDel marker

| **Cultivar name** | **Abbreviation** | **Leaf color** | **PCR amplification results of** **the leaf color InDel marker** |
| --- | --- | --- | --- |
| ‘Ebony Fire’ | BD1 | Purple leaves | Two bands (224 bp and 192 bp) |
| ‘Ebony Glow’ | BD2 | Purple leaves | Two bands (224 bp and 192 bp) |
| ‘Ebony Embers’ | BD4 | Purple leaves | Two bands (224 bp and 192 bp) |
| ‘Ebony and Ivory’ | BD5 | Purple leaves | Two bands (224 bp and 192 bp) |
| ‘Dynamite’ | BY | Young purple leaves and mature green leaves | One band (224 bp) |
| ‘Red Rocket’ | HHJ | Young purple leaves and mature green leaves | One band (224 bp) |
| ‘Whit III’ | TER | Young purple leaves and mature green leaves | One band (224 bp) |
| ‘Arapahoe’ | M54 | Green leaves | One band (224 bp) |
| ‘Pocomoke’ | M52 | Green leaves | One band (224 bp) |
| ‘Binfen Jiaren’ | BFJR | Green leaves | One band (224 bp) |
| ‘Fen Linglong’ | FLL | Green leaves | One band (224 bp) |
| ‘Fen Jingling’ | FJL | Green leaves | One band (224 bp) |
| ‘Ling Long’ | LL | Green leaves | One band (224 bp) |

Table S10 Physicochemical properties of three LiBBX16 protein

| Protein name | Number of amino acids | Molecular weight /kDa | Isoelectric point | Instability coefficient /% | Hydrophilicity |
| --- | --- | --- | --- | --- | --- |
| LiBBX16a | 249 | 27.67 | 4.77 | 51.33 | -0.416 |
| LiBBX16g | 249 | 27.66 | 4.81 | 50.96 | -0.418 |
| LiBBX16p | 221 | 24.60 | 5.20 | 47.25 | -0.394 |

Table S11 BBXs used for the phylogenetic analysis

| **Name** | **Species** | **Accession number/Reference** | **Group** |
| --- | --- | --- | --- |
| AtBBX1 | *Arabidopsis thaliana* | AT5G15840 | Ⅰ |
| AtBBX2 | *Arabidopsis thaliana* | AT5G15850 | Ⅰ |
| AtBBX3 | *Arabidopsis thaliana* | AT3G02380 | Ⅰ |
| AtBBX4 | *Arabidopsis thaliana* | AT2G24790 | Ⅰ |
| AtBBX5 | *Arabidopsis thaliana* | AT5G24930 | Ⅰ |
| AtBBX6 | *Arabidopsis thaliana* | AT5G57660 | Ⅰ |
| AtBBX7 | *Arabidopsis thaliana* | AT3G07650 | Ⅱ |
| AtBBX8 | *Arabidopsis thaliana* | AT5G48250 | Ⅱ |
| AtBBX9 | *Arabidopsis thaliana* | AT4G15250 | Ⅱ |
| AtBBX10 | *Arabidopsis thaliana* | AT3G21880 | Ⅱ |
| AtBBX11 | *Arabidopsis thaliana* | AT2G47890 | Ⅱ |
| AtBBX12 | *Arabidopsis thaliana* | AT2G33500 | Ⅱ |
| AtBBX13 | *Arabidopsis thaliana* | AT1G28050 | Ⅱ |
| AtBBX14 | *Arabidopsis thaliana* | AT1G68520 | Ⅲ |
| AtBBX15 | *Arabidopsis thaliana* | AT1G25440 | Ⅲ |
| AtBBX16 | *Arabidopsis thaliana* | AT1G73870 | Ⅲ |
| AtBBX17 | *Arabidopsis thaliana* | AT1G49130 | Ⅲ |
| AtBBX18 | *Arabidopsis thaliana* | AT2G21320 | Ⅳ |
| AtBBX19 | *Arabidopsis thaliana* | AT4G38960 | Ⅳ |
| AtBBX20 | *Arabidopsis thaliana* | AT4G39070 | Ⅳ |
| AtBBX21 | *Arabidopsis thaliana* | AT1G75540 | Ⅳ |
| AtBBX22 | *Arabidopsis thaliana* | AT1G78600 | Ⅳ |
| AtBBX23 | *Arabidopsis thaliana* | AT4G10240 | Ⅳ |
| AtBBX24 | *Arabidopsis thaliana* | AT1G06040 | Ⅳ |
| AtBBX25 | *Arabidopsis thaliana* | AT2G31380 | Ⅳ |
| AtBBX26 | *Arabidopsis thaliana* | AT1G60250 | Ⅴ |
| AtBBX27 | *Arabidopsis thaliana* | AT1G68190 | Ⅴ |
| AtBBX28 | *Arabidopsis thaliana* | AT4G27310 | Ⅴ |
| AtBBX29 | *Arabidopsis thaliana* | AT5G54470 | Ⅴ |
| AtBBX30 | *Arabidopsis thaliana* | AT4G15248 | Ⅴ |
| AtBBX31 | *Arabidopsis thaliana* | AT3G21890 | Ⅴ |
| AtBBX32 | *Arabidopsis thaliana* | AT3G21150 | Ⅴ |
| PpBBX24 | *Pyrus* spp. | (Ou et al., 2020) | Ⅳ |
| PpBBX24-del | *Pyrus* spp. | (Ou et al., 2020) | Ⅳ |
| GhDR | *Gossypium hirsutum* | (Wang et al., 2023) | Ⅳ |
| CmBBX24 | *Cucumis melo* | XP_008460038.1 | Ⅳ |
| OsBBX24 | *Oryza sativa* | XP_015636444.1 | Ⅳ |
| CmBBX24 | *Chrysanthemum x morifolium* | AIE76497.1 | Ⅳ |
| SsBBX24 | *Solanum sogarandinum* | ABC25454.1 | Ⅳ |
| MdCOL11 | *Malus domestica* | ADL36666.1 | Ⅳ |
| MdCOL4 | *Malus domestica* | ADL36673.1 | Ⅳ |
| PyBBX21 | *Pyrus x bretschneideri* | XP_009335579.2 | Ⅳ |
| PyBBX24L | *Pyrus ussuriensis x Pyrus communis* | KAB2604373.1 | Ⅳ |
| MdBBX10 | *Malus domestica* | XP_008364582.1 | Ⅴ |
| MdBBX20 | *Malus domestica* | RXH90160.1 | Ⅱ |
| MdBBX22 | *Malus domestica* | XP_008348545.3 | Ⅳ |
| MdBBX24 | *Malus domestica* | NP_001315848.1 | Ⅳ |
| MdBBX37 | *Malus domestica* | XP_028964037.2 | Ⅴ |
| MdBBX43 | *Malus domestica* | XP_008341828.3 | Ⅰ |

Table S12 Downstream targeted structural genes of *LiBBX16p* in anthocyanin biosynthesis identified by KEGG analysis

| Gene ID | Symbol | Ko Number | NR Annotation |
| --- | --- | --- | --- |
| Lin_chr2_0875 | ACT | K13065 | vinorine synthase-like [*Punica granatum*] |
| Lin_chr7_1625 | CYP98A2 | K09754 | cytochrome P450 98A2 [*Punica granatum*] |
| Lin_chr19_0678 | HHT1 | K13065 | fatty alcohol: caffeoyl-CoA acyltransferase [*Punica granatum*] |
| Lin_chr9_0835 | CHI | K01859 | chalcone--flavonone isomerase-like [*Punica granatum*] |
| Lin_chr20_0230 | F3H | K00475 | naringenin,2-oxoglutarate 3-dioxygenase [*Punica granatum*] |
| Lin_chr14_0817 | CYP73A4 | K00487 | hypothetical protein CDL15_Pgr010283 [*Punica granatum*] |
| Lin_chr22_0293 | CYP75B1 | K05280 | flavonoid 3&apos; -monooxygenase [*Punica granatum*] |
| Lin_chr22_0291 | CYP75B2 | K05280 | flavonoid 3&apos; -monooxygenase [*Punica granatum*] |
| Lin_chr19_0390 | BAHD1 | K13065 | BAHD acyltransferase At5g47980-like [*Punica granatum*] |
| Lin_chr16_0223 | - | K00660 | chalcone synthase 3-like [*Punica granatum*] |
| Lin_chr18_0557 | CYP73A13 | K00487 | hypothetical protein CDL15_Pgr010283 [*Punica granatum*] |
| Lin_chr5_0628 | CHS | K00660 | chalcone synthase 3-like [*Punica granatum*] |
| Lin_chr21_0099 | - | K08695 | anthocyanidin reductase ((2S)-flavan-3-ol-forming) isoform X2 [*Punica granatum*] |
| Lin_chr14_0578 | F3GT | K12930 | flavonoid 3-O-glucosyltransferase-like [*Punica granatum*] |

Table S13 Downstream target transcription factors of *LIBBX16p* in anthocyanin biosynthesis identified by RNA-Seq and Dap-seq data

| Gene ID | Symbol | NR Annotation |
| --- | --- | --- |
| Lin_chr9_0835 | MYB75 | transcription factor MYB75-like [*Punica granatum*] |
| Lin_chr19_0277 | MYB5 | transcription factor MYB5-like [*Punica granatum*] |

Table S14 MYB transcription factors used for the phylogenetic analysis

| **Name** | **Species** | **Accession number** | **Functional category** |
| --- | --- | --- | --- |
| AtMYB123 | *Arabidopsis thaliana* | CAC40021 | Proanthocyanidin activator |
| FaMYB9 | *Fragaria × ananassa* | AFL02460 | Proanthocyanidin activator |
| FaMYB11 | *Fragaria × ananassa* | AFL02461 | Proanthocyanidin activator |
| MdMYB11 | *Malus × domestica* | AAZ20431 | Proanthocyanidin activator |
| AtMYB5 | *Arabidopsis thaliana* | AEE75369 | Flavonoid activator |
| VvMYB5a | *Vitis vinifera* | AAS68190 | Flavonoid activator |
| VvMYB5b | *Vitis vinifera* | AAX51291 | Flavonoid activator |
| AtMYB11 | *Arabidopsis thaliana* | AEE80369 | Flavonol activator |
| AtMYB12 | *Arabidopsis thaliana* | AEC10843 | Flavonol activator |
| AtMYB111 | *Arabidopsis thaliana* | AED95797 | Flavonol activator |
| PhMYB27 | *Petunia × hybrida* | AHX24372 | Anthocyanin represser |
| AtMYB3 | *Arabidopsis thaliana* | AEE30263 | Anthocyanin represser |
| AtMYB6 | *Arabidopsis thaliana* | NP_192684 | Anthocyanin represser |
| NtMYB2 | *Narcissus tazetta* | ATO58377 | Anthocyanin represser |
| AtMYB7 | *Arabidopsis thaliana* | NP_179263 | Anthocyanin represser |
| AtMYB4 | *Arabidopsis thaliana* | AEE86955 | Anthocyanin represser |
| LhMYB12 | *Lilium hybrid* | BAJ05398 | Anthocyanin activator |
| LhMYB3 | *Lilium hybrid* | BAJ05399 | Anthocyanin activator |
| MlMYB1 | *Magnolia liliiflora* | AHJ60260 | Anthocyanin activator |
| FaMYB10 | *Fragaria × ananassa* | ABX79947 | Anthocyanin activator |
| MdMYB1 | *Malus × domestica* | ABK58136 | Anthocyanin activator |
| MdMYB10 | *Malus × domestica* | ABB84753 | Anthocyanin activator |
| PgMYB | *Punica granatum* | AHZ97876 | Anthocyanin activator |
| VvMYBA3 | *Vitis vinifera* | ABL14067 | Anthocyanin activator |
| VvMYBA2 | *Vitis vinifera* | BAD18978 | Anthocyanin activator |
| AtMYB113 | *Arabidopsis thaliana* | AEE34501 | Anthocyanin activator |
| AtMYB75 | *Arabidopsis thaliana* | AAG42001 | Anthocyanin activator |
| AtMYB90 | *Arabidopsis thaliana* | AAG42002 | Anthocyanin activator |
| AmVENOSA | *Antirrhinum majus* | ABB83828 | Anthocyanin activator |
| AmROSEA1 | *Antirrhinum majus* | ABB83826 | Anthocyanin activator |
| SlAN1 | *Solanum lycopersicum* | NP_001234417 | Anthocyanin activator |
| PhDPL | *Petunia × hybrida* | ADQ00395 | Anthocyanin activator |
| PhAN4 | *Petunia × hybrida* | ADQ00392 | Anthocyanin activator |
| PhAN2 | *Petunia × hybrida* | AAF66727 | Anthocyanin activator |
| CaA | *Capsicum annuum* | CAE75745 | Anthocyanin activator |
| PhPHZ | *Petunia × hybrida* | ADQ00390 | Anthocyanin activator |

Table S15 Glycosyltransferases used for the phylogenetic analysis

| **Name** | **Species** | **Accession number** | **Group** |
| --- | --- | --- | --- |
| AcA3GalT | *Aralia cordata* | BAD06514 | F |
| BpAGAT | *Bellis perennis* | BAD77944 | A |
| Cm1,2RT | *Citrus maxima* | AAL06646 | A |
| CuLGT | *Citrus unshiu* | BAA93039 | L |
| DkFGT | *Diospyros kaki* | AB435084^1^ | F |
| GtAGT | *Gentiana triflora* | BAC54092 | D |
| In3GGT | *Ipomoea nil* | BAD95883 | A |
| PhF3GalT | *Petunia x hybrida* | Q9SBQ8 | F |
| PhRT | *Petunia x hybrida* | Q43716 | A |
| RhGT1 | *Rosa hybrid cultivar* | Q4R1I9 | E |
| MtUGT72L1 | *Medicago truncatula* | ACC38470 | E |
| AtUGT73B2 | *Arabidopsis thaliana* | At4g34135 | D |
| AtUGT73C5 | *Arabidopsis thaliana* | At2g36800 | D |
| AtUGT73C6 | *Arabidopsis thaliana* | At2g36790 | D |
| AtUGT75C1 | *Arabidopsis thaliana* | At4g14090 | L |
| AtUGT75D1 | *Arabidopsis thaliana* | At4g15550 | L |
| MtUGT75L4 | *Maclura pomifera* | ABL85474 | L |
| AtUGT76C1 | *Arabidopsis thaliana* | At5g05870 | H |
| AtUGT78D1 | *Arabidopsis thaliana* | At1g30530 | F |
| AtUGT78D2 | *Arabidopsis thaliana* | At5g17050 | F |
| AtUGT78D3 | *Arabidopsis thaliana* | At5g17030 | F |
| AtUGT79B1 | *Arabidopsis thaliana* | At5g54060 | A |
| AtUGT82A1 | *Arabidopsis thaliana* | At3g22250 | N |
| AtUGT83A1 | *Arabidopsis thaliana* | At3g02100 | I |
| AtUGT85A1 | *Arabidopsis thaliana* | At1g22400 | G |
| AtUGT86A1 | *Arabidopsis thaliana* | At2g36970 | K |
| AtUGT87A1 | *Arabidopsis thaliana* | At2g30150 | J |
| MtUGT88A4 | *Maclura pomifera* | ABL85471 | E |
| AtUGT89B1 | *Arabidopsis thaliana* | At1g73880 | B |
| AtUGT90A2 | *Arabidopsis thaliana* | At1g10400 | C |
| AtUGT92A1 | *Arabidopsis thaliana* | At5g12890 | M |
| Vm3GalT | *Vigna mungo* | BAA36972 | F |
| Dc3GT | *Dianthus aryophyllus* | BAD52003 | F |
| Dk3GT | *Diospyros kaki* | BAI40148 | F |
| Fa3GT | *Fragaria x ananassa* | Q66PF5.1 | F |
| Fi3GT | *Forsythia x intermedia* | AAD21086 | F |
| Mt3GT | *Medicago truncatula* | XP_003610163 | F |
| Pf3GT | *Perilla frutescens* | BAA19659 | F |
| Fh3GT1 | *Fressia hybrida* | ADK75021.1 | F |
| Gt3GT | *Gentiana triflora* | BAA12737.1 | F |
| Hv3GT | *Hordeum vulgare* | CAA33729 | F |
| Ih3GT | *Iris hollandica* | BAD83701 | F |
| PhUF3GT | *Petunia hybrida* | AAD55985 | F |
| VlFd3GT | *Vitis labrusca* | ABR24135 | F |
| Sm3GT | *Solanum melongena* | Q43641 | F |
| VvUF3GT | *Vitis vinifera* | AAB81683 | F |
| Zm3GT | *Zea mays* | CAA31856 | F |
| Gh5GT | *Glandularia x hybrida* | BAA36423 | L |
| Th5GT | *Torenia hybrid* | BAC54093 | L |
| Gt5GT | *Gentiana triflora* | BAG32255 | L |
| Ih5GT | *Iris hollandica* | BAD06874 | L |
| Pf5GT1 | *Perilla frutescens* | BAA36421 | L |
| Pf5GT2 | *Perilla frutescens* | BAA36422 | L |
| PhUF5GT | *Petunia hybrida* | BAA89009 | L |
| Ac73G1 | *Allium cepa* | AAP88406 | D |
| Sb7GT | *Scutellaria baicalensis* | BAA83484 | D |
| GmIsoflv7GT | *Glycine max* | ABB85236 | D |

Table S16 Leaf color and anthocyanin contents of the ‘Arapahoe’ × ‘Ebony Embers’ population.

| **Progeny ID** | **Visual Inspection** | **Color Number** | | **Anthocyanin Content（mg/g）** |
| --- | --- | --- | --- | --- |
| 1 | Purple | GREYED PURPLE | N186C | 5.11 |
| 2 | Greyish-purple | GREYED PURPLE | N187B | 6.28 |
| 3 | Dark green | GREEN | 139C | 0.59 |
| 4 | Greyish-purple | GREYED PURPLE | N187A | 3.81 |
| 5 | Purple | PURPLE | N77A | 4.70 |
| 6 | Greyish-purple | GREYED PURPLE | N189C | 6.24 |
| 7 | Greyish-green | GREYED GREEN | 144C | 0.64 |
| 8 | Green | GREEN | 141A | 0.57 |
| 9 | Purple | PURPLE | N77A | 4.70 |
| 10 | Purple | GREYED PURPLE | N187B | 5.86 |
| 11 | Greyish-purple | GREYED PURPLE | N187B | 5.24 |
| 12 | Greyish-purple | GREYED PURPLE | N189A | 1.89 |
| 13 | Purple | GREYED PURPLE | N189C | 2.60 |
| 14 | Greyish-purple | GREYED PURPLE | N186B | 3.55 |
| 15 | Greyish-purple | GREYED PURPLE | N189C | 4.66 |
| 16 | Dark purple | GREYED PURPLE | 187A | 4.89 |
| 17 | Purple | PURPLE | N77A | 4.70 |
| 18 | Greenish-purple | GREYED GREEN | N189B | 3.50 |
| 19 | Purple | GREYED PURPLE | N186A | 4.09 |
| 20 | Greenish-purple | GREYED PURPLE | 187A | 2.81 |
| 21 | Greyish-purple | GREYED PURPLE | N189C | 5.19 |
| 22 | Greenish-purple | PURPLE | N77A | 4.22 |
| 23 | Purple | GREYED PURPLE | N186A | 2.61 |
| 24 | Green | GREEN | 141C | 0.72 |
| 25 | Green | GREEN | 143B | 0.37 |
| 26 | Greenish-purple | PURPLE | N77A | 4.35 |
| 27 | Dark green | GREEN | 139A | 0.56 |
| 28 | Purple | GREYED PURPLE | N189B | 2.98 |
| 29 | Purple | GREYED PURPLE | N189A | 5.06 |
| 30 | Purple | GREYED PURPLE | N187B | 5.31 |
| 31 | Purple | GREYED PURPLE | N186A | 4.10 |
| 32 | Purple | GREYED PURPLE | N186A | 3.46 |
| 33 | Greyish-green | GREYED GREEN | 189A | 0.63 |
| 34 | Green | GREEN | 141B | 0.76 |
| 35 | Green | GREEN | 143A | 0.41 |
| 36 | Green | GREEN | 141B | 0.30 |
| 37 | Green | GREEN | N134A | 0.58 |
| 39 | Greyish-purple | GREYED PURPLE | 147A | 2.19 |
| 40 | Green | GREEN | 143A | 0.43 |
| 41 | Green | GREEN | 138B | 0.51 |
| 42 | Green | GREEN | 136A | 0.53 |
| 43 | Green | GREEN | 143A | 0.26 |
| 44 | Green | GREEN | 135C | 0.51 |
| 45 | Purple | GREYED PURPLE | N186A | 4.00 |
| 46 | Green | GREEN | 143A | 0.31 |
| 47 | Green | GREEN | 135B | 0.43 |
| 48 | Green | GREEN | 141B | 0.33 |
| 49 | Green | GREEN | 139A | 0.35 |
| 50 | Green | GREEN | 136C | 0.34 |
| 51 | Greyish-purple | GREYED PURPLE | N137A | 2.08 |
| 52 | Purple | GREYED PURPLE | N186A | 3.36 |
| 53 | Greyish-purple | GREYED PURPLE | N189C | 3.23 |
| 54 | Green | GREEN | 141B | 0.68 |
| 55 | Purple | GREYED GREEN | N189B | 1.11 |
| 56 | Purple | GREYED PURPLE | N187C | 2.31 |
| 57 | Green | GREEN | 141B | 0.46 |
| 58 | Green | GREEN | 141B | 0.59 |
| 59 | Green | GREEN | 143A | 0.32 |
| 60 | Green | GREEN | 139A | 0.49 |
| 61 | Greyish-purple | GREYED PURPLE | N189C | 4.72 |
| 62 | Purple | GREYED PURPLE | N186A | 4.22 |
| 63 | Green | GREEN | 143A | 0.33 |
| 64 | Green | GREEN | 141C | 0.48 |
| 65 | Green | GREEN | N134A | 0.27 |
| 66 | Green | GREEN | 143A | 0.29 |
| 67 | Green | GREEN | 141B | 0.32 |
| 68 | Green | GREEN | 141A | 0.44 |
| 69 | Green | GREEN | 143A | 0.33 |
| 70 | Green | GREEN | 143A | 0.30 |
| 71 | Greyish-purple | PURPLE | N77A | 4.32 |
| 72 | Purple | GREYED PURPLE | N186A | 3.60 |
| 73 | Purple | GREYED PURPLE | N186A | 2.67 |
| 74 | Greyish-purple | GREEN | 136B | 2.21 |
| 75 | Green | GREEN | 141C | 0.43 |
| 76 | Greyish-purple | GREYED PURPLE | N186A | 4.37 |
| 77 | Purple | GREYED PURPLE | N186C | 5.25 |
| 79 | Green | GREEN | 143B | 0.52 |
| 80 | Green | GREEN | 139A | 0.32 |
| 114 | Purple | GREYED PURPLE | N186A | 4.30 |
| ‘Arapahoe’ | Green | GREEN | N134A | 0.30 |
| ‘Ebony Embers’ | Greyish-purple | GREYED PURPLE | N186A | 4.86 |

Table S17 List of all primer sequences.

| **Primer name** | **Primer sequence (5' to 3')** | **Purpose** |
| --- | --- | --- |
| LiLDOX-cDNA-F | GCTTCACACTTCTTCTTCGGTGGT | Gene cloning |
| LiLDOX-cDNA-R | TCATTTGGATTTCTCCTCTGGGTTATTCC |  |
| LiBBX16-cDNA-F | ATGAAGATACAGTGCGATGTCTGC |  |
| LiBBX16-cDNA-R | TCAATCTAAGCCGAGGTCGGGGACAG |  |
| LiBBX16-gDNA-F | ATGAAGATACAGTGCGATGTCTGC |  |
| LiBBX16-gDNA-R | TCAATCTAAGCCGAGGTCGGGGACAG |  |
| LiLDOXpro-F | CCTACCGCAACCCACTAT | Promoter cloning |
| LiLDOXpro-R | CTTCTTCTCAAAACCCTAATGC |  |
| LiBBX16pro-F | GGACTCTCTCTTCTCCCT |  |
| LiBBX16pro-R | TCGCACTGTATCTTCATC |  |
| 1300-LiBBX16p-F | TCGACTCTAGTCTAGAATGAAGATACAGTGCGATGTCTGC | Vector construction |
| 1300-LiBBX16p-R | CCCTTGCTCACCATGGTACCTATGCAGGGATTTCGATCCTCTG |  |
| 1300-LiBBX16g-F | TCGACTCTAGTCTAGAATGAAGATACAGTGCGATGTCTGCG |  |
| 1300-LiBBX16g-R | CCCTTGCTCACCATGGTACCATCTAAGCCGAGGTCGGGG |  |
| 1300-LiBBX16a-F | TCGACTCTAGTCTAGAATGAAGATACAGTGCGATGTCTGCG |  |
| 1300-LiBBX16a-R | CCCTTGCTCACCATGGTACCATCTAAGCCGAGGTCGGGG |  |
| 1300-F | TTACCAATACATTACACTAGCATCTG | Positive identification |
| 1300-R | CTGAACTTGTGGCCGTTT |  |
| pTRV2-LiBBX16-F | TAAGGTTACCGAATTCGAATCCGAGTCGCGTTAAGCTC | Vector construction |
| pTRV2-LiBBX16-R | GCTCGGTACCGGATCCCCATGTCAGCTAGCCAGTC |  |
| pTRV2-F | TGTTTGAGGGAAAAGTAGAGAACGT | Positive identification |
| pTRV2-R | TTACCGATCAATCAAGATCAGTCGA |  |
| pTRV1-F | TTACAGGTTATTTGGGCTAG |  |
| pTRV1-R | CCGGGTTCAATTCCTTATC |  |
| LiCHS-qRT-F | GCGAGGCAGGACATAGTGGT | qRT-PCR |
| LiCHS-qRT-R | GAGGTGGTGCAGAAGATGAGGT |  |
| LiCHI-qRT-F | GGTGACTGAGAACTGCGTGGCT |  |
| LiCHI-qRT-R | CCCGTGCTTCCCGATGA |  |
| LiF3H-qRT-F | TGTCCGTAGCGACGTTCCA |  |
| LiF3H-qRT-R | TCCTTGCTCATCTTCCTCCTGT |  |
| LiF3’H-qRT-F | GTGGAGGCGAAGATGAAGAA |  |
| LiF3’H-qRT-R | TCTGTCCCTGCCGAGAA |  |
| LiDFR-qRT-F | CCAAGACACTGGCAGAGCA |  |
| LiDFR-qRT-R | CATCGACGGCATCAGGAA |  |
| LiBBX16-qRT-F | CAGCAGCAGGATTCTCTATC |  |
| LiBBX16-qRT-R | CCTTCTTGTCAGAGGAGTTG |  |
| LiMYB75-qRT-F | CGTCATAAAGCCTCGTCCTC |  |
| LiMYB75-qRT-R | GGTGGCGCTGTAGTCTAAC |  |
| LiHY5-qRT-F | GAGCCCTGCAGATAAAGAGAAC |  |
| LiHY5-qRT-R | CTGCCTGCTGTAGTGTTCTT |  |
| EF-α-F | GACTGTGCTGTGCTCATC |  |
| EF-α-R | GTGGCATCCATCTTGTTG |  |
| LiCYP73A4pro-F | GATTCTCATTCCACGAGGGGAAGA | Promoter cloning |
| LiCYP73A4pro-R | GTACTACTGCTACACCTTGTCCCT |  |
| LiCYP73A13pro-F | AGGGAGGTTTGACGAAGCAAATGT |  |
| LiCYP73A13pro-R | CTATCACATGCCTACAAATC |  |
| LiCHIpro-F | GAAGACAAAGATAGTTGGTCTCAC |  |
| LiCHIpro-R | GGTGGGACGTTTCGTTC |  |
| LiF3Hpro-F | CAATCACCCGACCATTAGGTAG |  |
| LiF3Hpro-R | GCTGAACTCGTTGTAGGCGACCTT |  |
| LiF3GTpro-F | TCACTCAGTCCTAATTGTAGCCT |  |
| LiF3GTpro-R | GTTGAACTGGTTGGAACTTA |  |
| LiMYB5pro-F | GAACAGATGGAACAGGGACAC |  |
| LiMYB5pro-R | GCCCAATCCAAAAGCTAGATA |  |
| LiMYB75pro-F | CCGCTCCCATATTTATTTGC |  |
| LiMYB75pro-R | TGTTATTCCTCACAAGTAGGTGG |  |
| SK-LiBBX16p-F | GGACAGCCCAAGCTGAGCTCATGAAGATACAGTGCGATGTCTGC | Vector construction |
| SK-LiBBX16p-R | CAGCGTACCGAATTGGTACCTCATATGCAGGGATTTCGATCC |  |
| SK-LiBBX16g-F | GGACAGCCCAAGCTGAGCTCATGAAGATACAGTGCGATGTCTG |  |
| SK-LiBBX16g-R | CAGCGTACCGAATTGGTACCTCAATCTAAGCCGAGGTCG |  |
| LUC-CYP73A4-F | GGGCGAATTGGGTACCGATTCTCATTCCACGAGGGGAA |  |
| LUC-CYP73A4-R | GCTCTAGAACTAGTGGATCCGTACTACTGCTACACCTTGTC |  |
| LUC-CYP73A13-F | GGGCGAATTGGGTACCTATCACATGCCTACAAATCAACAAA |  |
| LUC-CYP73A13-R | GCTCTAGAACTAGTGGATCCGTTACTCTCACTCTGGTATTATA |  |
| LUC-pCHI-F | GGGCGAATTGGGTACCTGAAGACAAAGATAGTTGGTCTCAC |  |
| LUC-pCHI-R | GCTCTAGAACTAGTGGATCCGGAATGGGCTCTGGCGAG |  |
| LUC-pF3H-F | GGGCGAATTGGGTACCTCAATCACCCGACCATTAGGTA |  |
| LUC-pF3H-R | GCTCTAGAACTAGTGGATCCAGTGCTGGTTATCAATGTGGGATTT |  |
| LUC-pF3GT-F | GGGCGAATTGGGTACCTCACTCAGTCCTAATTGTAGCCTA |  |
| LUC-pF3GT-R | GCTCTAGAACTAGTGGATCCCGAAGATTCACTGCATAATGTAGTC |  |
| LUC-pMYB5-F | GGGCGAATTGGGTACCGAACAGATGGAACAGGGACG |  |
| LUC-pMYB5-R | GCTCTAGAACTAGTGGATCCGCCCAATCCAAAAGCTAGATATC |  |
| LUC-pMYB75-F | GGGCGAATTGGGTACCATTCCTGCGGTGTTATCGGAAAT |  |
| LUC-pMYB75-R | GCTCTAGAACTAGTGGATCCGTTATTCCTCACAAGTAGGTGGAA |  |
| 0800-F | CAAGGCGATTAAGTTGGGT | Positive identification |
| 0800-R | AGGAACCAGGGCGTATCT |  |
| 62SK-F | GATGACGCACAATCCCACTAT |  |
| 62SK-R | AGCGAAACCCTATAAGAACCCT |  |
| BD-LiBBX16g-F | CATGGAGGCCGAATTCATGAAGATACAGTGCGATGTCTGCG | Vector construction |
| BD-LiBBX16g-R | GCAGGTCGACGGATCCATCTAAGCCGAGGTCGGGG |  |
| BD-LiBBX16p-F | CATGGAGGCCGAATTCATGAAGATACAGTGCGATGTCTGC |  |
| BD-LiBBX16p-R | GCAGGTCGACGGATCCTATGCAGGGATTTCGATCCTCTG |  |
| BD-LiBBX16-N-F | CATGGAGGCCGAATTCATGAAGATACAGTGCGATGTCTGCG |  |
| BD-LiBBX16-N-R | GCAGGTCGACGGATCCGAGAAACCTCTGGTGGTTGGC |  |
| BD-LiBBX16g-C-F | CATGGAGGCCGAATTCATGGCGACCGGAATCCGA |  |
| BD-LiBBX16g-C-R | GCAGGTCGACGGATCCATCTAAGCCGAGGTCGGGGA |  |
| BD-LiBBX16p-C-F | CATGGAGGCCGAATTCATGGCGACCGGAATCCGA |  |
| BD-LiBBX16p-C-R | GCAGGTCGACGGATCCTATGCAGGGATTTCGATCCTCTGT |  |
| AD-F | CGCCATGGAGTACCCATAC | Positive identification |
| AD-R | TACGATTCATCTGCAGCTCG |  |
| BD-F | CGCCGGAATTTGTAATACGA |  |
| BD-R | GAAATTCGCCCGGAATTAGC |  |
| LiF3GT-F | ATGTCAGGACTGTCCGAAGCAT | Gene cloning |
| LiF3GT-R | GAGTCTCCATTACCTACGAG |  |
| LiMYB5-F | ATGAGGAGCCCGAGAACG |  |
| LiMYB5-R | CTATTCAGTGTGATCGTTGCTGTAA |  |
| LiMYB75-F | ATGGAGGCACCTTCAGGAGTG |  |
| LiMYB75-R | TTATCCAATTAATTCACTGACGCC |  |
| 1300-LiF3GT-F | TCGACTCTAGTCTAGAATGTCAGGACTGTCCGAAGCA | Vector construction |
| 1300-LiF3GT-R | CCCTTGCTCACCATGGTACCTTTTTTGTCCACGATCTCCATCA |  |
| 1300-LiMYB5-F | CAAATCGACTCTAGTCTAGAATGAGGAGCCCGAGAACG |  |
| 1300-LiMYB5-R | GACGTAACATGGTACCTTCAGTGTGATCGTTGCTGTAATCA |  |
| 1300-LiMYB75-F | TCGACTCTAGTCTAGAATGGAGGCACCTTCAGGAGTG |  |
| 1300-LiMYB75-R | CCCTTGCTCACCATGGTACCTCCAATTAATTCACTGACGCC |  |

Note: Primers containing an underline indicate that the restriction site recognizes the base.
